# Neurite dispersion: a new marker of multiple sclerosis spinal cord pathology?

**DOI:** 10.1002/acn3.445

**Published:** 2017-08-15

**Authors:** Francesco Grussu, Torben Schneider, Carmen Tur, Richard L. Yates, Mohamed Tachrount, Andrada Ianuş, Marios C. Yiannakas, Jia Newcombe, Hui Zhang, Daniel C. Alexander, Gabriele C. DeLuca, Claudia A. M. Gandini Wheeler‐Kingshott

**Affiliations:** ^1^ NMR Research Unit Department of Neuroinflammation Queen Square MS Centre UCL Institute of Neurology University College London London United Kingdom; ^2^ Centre for Medical Image Computing Department of Computer Science University College London London United Kingdom; ^3^ Philips UK Guildford Surrey United Kingdom; ^4^ Nuffield Department of Clinical Neurosciences University of Oxford Oxford United Kingdom; ^5^ Department of Brain Repair and Rehabilitation UCL Institute of Neurology University College London London United Kingdom; ^6^ NeuroResource UCL Institute of Neurology University College London London United Kingdom; ^7^ Brain MRI 3T Mondino Research Centre C. Mondino National Neurological Institute Pavia Italy; ^8^ Department of Brain and Behavioural Sciences University of Pavia Pavia Italy

## Abstract

**Objective:**

Conventional magnetic resonance imaging (MRI) of the multiple sclerosis spinal cord is limited by low specificity regarding the underlying pathological processes, and new MRI metrics assessing microscopic damage are required. We aim to show for the first time that *neurite orientation dispersion* (i.e., variability in axon/dendrite orientations) is a new biomarker that uncovers previously undetected layers of complexity of multiple sclerosis spinal cord pathology. Also, we validate against histology a clinically viable MRI technique for dispersion measurement (*neurite orientation dispersion and density imaging,* NODDI), to demonstrate the strong potential of the new marker.

**Methods:**

We related quantitative metrics from histology and MRI in four post mortem spinal cord specimens (two controls; two progressive multiple sclerosis cases). The samples were scanned at high field, obtaining maps of neurite density and orientation dispersion from NODDI and routine *diffusion tensor imaging* (DTI) indices. Histological procedures provided markers of astrocyte, microglia, myelin and neurofilament density, as well as neurite dispersion.

**Results:**

We report from both NODDI and histology a trend toward lower neurite dispersion in demyelinated lesions, indicative of reduced neurite architecture complexity. Also, we provide unequivocal evidence that NODDI‐derived dispersion matches its histological counterpart (*P* < 0.001), while DTI metrics are less specific and influenced by several biophysical substrates.

**Interpretation:**

Neurite orientation dispersion detects a previously undescribed and potentially relevant layer of microstructural complexity of multiple sclerosis spinal cord pathology. Clinically feasible techniques such as NODDI may play a key role in clinical trial and practice settings, as they provide histologically meaningful dispersion indices.

## Introduction

Multiple sclerosis is a central nervous system disease characterized by a complex pathophysiology,^1^ the fundamental features of which have been derived from the analysis of post mortem tissue.^2^, ^3^, ^4^, ^5^ Spinal cord pathology is an important determinant of permanent neurological disability with cardinal characteristics being inflammatory demyelination, synaptic, neuronal and axonal loss.^6^, ^7^, ^8^, ^9^, ^10^, ^11^ The precise nature of how these pathological alterations impact clinical phenotypes remains unknown.

Conventional magnetic resonance imaging (MRI) provides the unique opportunity to decipher the impact of multiple sclerosis spinal cord pathology on clinical outcomes in vivo. However, the pathological substrate of radiological changes are relatively nonspecific and fail to consistently correlate with measures of disability.^12^ New quantitative MRI methods that assess functionally relevant microstructure noninvasively and relate to changes at the microscopic level are urgently needed.

Quantitative MRI offers promising metrics that may provide higher specificity and sensitivity to early and diffuse microscopic damage,^13^ potentially overcoming the radiological‐clinical paradox^14^ (i.e., the poor association between clinical findings and extent of radiological involvement). In vivo mapping of myelin density^15^ and g‐ratio^16^; iron deposition^17^; sodium concentration^18^; inflammation and axonal injury^19^ are examples of quantitative MRI techniques being developed to detect early multiple sclerosis pathology. Similar quantitative MRI metrics require extensive validation, in order to confirm their specificity and relevance to clinical outcomes.^20^, ^21^, ^22^^.^

A heretofore neglected but potentially functionally relevant morphological feature of neural tissue is the complexity of the orientations of axons and dendrites, collectively known as neurites. Neurites allow the exchange of information among neurons, and there is evidence to believe that changes of their architecture can impact directly on neural function.^23^ Alteration of dendrite morphology could account *per se* for impairment beyond mere neuronal death.^24^ In multiple sclerosis, primary morphological changes have been recently detected at the level of the dendritic spines and branches.^25^ These findings suggest that mapping neurite morphology in multiple sclerosis is of high interest, as it can potentially provide novel insights about pathophysiology and better explain motor, sensory and cognitive deficits often encountered in the disease.

In this study, we aim to show that *neurite orientation dispersion*, namely the variability of neurite orientations, is a new sensitive and specific biomarker of multiple sclerosis spinal cord pathology. Furthermore, we aim to validate *neurite orientation dispersion and density imaging* (NODDI), a recent model‐based diffusion MRI technique.^26^ The clinical feasibility of NODDI in vivo has already been demonstrated,^26^, ^27^, ^28^, ^29^ and here we compare NODDI dispersion indices against histology to demonstrate that the new marker has strong clinical potential. NODDI belongs to the family of quantitative MRI methods, and unlike conventional radiographic readouts its metrics are designed to measure features of tissue microstructure at a length scale that is orders of magnitude smaller than the size of the imaging voxels. Through the combination of state‐of‐the‐art quantitative histology with post mortem NODDI imaging, we measure for the first time a trend toward reduced orientation dispersion (i.e., reduced geometrical complexity of neurite architecture) in multiple sclerosis demyelinated lesions. Moreover, we demonstrate that NODDI orientation dispersion index is histologically meaningful. Our work suggests that neurite dispersion characterizes the complexity of pathology at a new microstructural level that may provide important insight into the pathophysiological processes driving irreversible disability in multiple sclerosis.

## Materials and Methods

### Specimens

Formalin‐fixed post mortem tissue from the upper thoracic and upper lumbar spinal cord of two nonneurological disease controls (thoracic: 66‐year‐old male, length: 3.1 cm; lumbar: 67‐year‐old female, length: 2.1 cm) and two multiple sclerosis (thoracic: 75‐year‐old male, decease secondary to primary progressive multiple sclerosis, length: 3.3 cm; lumbar: 67‐year‐old female, decease secondary to secondary progressive multiple sclerosis, length: 2.4 cm) tissue donors was used (samples obtained from Oxford Brain Bank and UCL NeuroResource tissue bank). Donation followed written informed consent and the material was processed according to Human Tissue Authority guidelines, after Ethical Approvals by appropriate review boards. Samples were stored in 10% formalin and washed for 24 h in 10 mmol/L phosphate buffered saline solution for downstream imaging. Subsequently, each sample was cut in the mid‐sagittal plane to allow for accurate MRI‐histology comparison. Data S1 reports additional demographical details and technical information on our methodology.

### MRI acquisition

All specimens were scanned on a 9.4 T Agilent small animal scanner using a 33 mm surface coil and a 1 T m^−1^ gradient insert (temperature held constant at 35°C using a temperature probe and an MRI‐compatible air heater).

Structural images were acquired axially using a multi‐slice spin echo sequence, to define specific radiographic positions for the subsequent histological procedures (resolution 0.20 × 0.10 × 2.00 mm^3^, TE/TR = 20/614 msec). Twenty 0.800 mm‐thick diffusion‐weighted slices were acquired sagittally, parallel to the surface exposed by the mid‐sagittal cut, using a multi‐slice spin echo sequence (resolution: 0.16 × 0.20 mm^2^; field‐of‐view: 21 × 51.2 mm^2^; TE/TR = 39.5/2200 msec; *δ*/Δ = 12/18 msec; 25 *b* = 0 images; six *b*‐shells: *b* of 520, 2080, 4680, 8320, 13,000, 18,720 sec mm^−2^ with 6, 15, 24, 33, 42, 51 isotropically distributed gradient directions).

### MRI signal analysis

The NODDI model was fitted to the diffusion data voxel‐by‐voxel in Matlab (The MathWorks, Inc., Natick, MA) with the NODDI toolbox (http://nitrc.org/projects/noddi_toolbox), setting the free water diffusivity to that of the buffer solution and the intrinsic diffusivity of the neural tissue to 1.50 *μ*m^2^ msec^−1^ (as this value maximizes the quality of the fit in all specimens). An additional compartment of isotropic restriction was employed, as in other ex vivo studies.^30^

Model fitting provided maps of isotropic volume fraction (IVF), neurite density index (NDI) and orientation dispersion index (ODI) (Table 1). IVF is the volume fraction of the water pool characterized by isotropic diffusion, and can be interpreted as the voxel volume fraction of free water, in this instance the buffer solution (cerebrospinal fluid in vivo). NDI describes the volume fraction of zero‐radius cylinders, designed to describe axons and dendrite. Therefore, it models the amount of neurites within a voxels. ODI is obtained fitting a Watson distribution to the zero‐radius cylinder orientation distribution^26^, and is a metric designed to characterize neurite orientation variability, ranging from 0 (axon/dendrites all parallel) to 1 (axon/dendrites isotropically randomly oriented).

**Table 1:** Summary and description of NODDI and histological metrics

Metric name	Abbreviation	Modality	Description
Orientation dispersion index	ODI	NODDI	Variability of neurite orientations
Neurite density index	NDI	NODDI	Amount of neurites
Isotropic volume fraction	IVF	NODDI	Amount of free water
Fractional anisotropy	FA	DTI	Anisotropy of diffusion profile
Axial diffusivity	AD	DTI	Diffusivity along principal tensor direction
Radial diffusivity	RD	DTI	Diffusivity across principal tensor direction
Mean diffusivity	MD	DTI	Mean rate of diffusion
Circular variance	CV	Histology	Variability of neurite orientations
Myelin staining fraction	MSF	Histology	Amount of myelin
Neurofilament staining fraction	NSF	Histology	Amount of neurofilaments
Astrocyte staining fraction	ASF	Histology	Amount of astrocytes
Microglia staining fraction	*μ*GSF	Histology	Amount of microglia

For comparison, we also fitted the standard diffusion tenor imaging (DTI) model^31^ to the measurements in Gaussian diffusion regime (up to *b* = 4680 sec mm^−2^) using in‐house Matlab code. From the fitted tensor, fractional anisotropy (FA), axial, radial, and mean diffusivities (AD, RD, and MD) were obtained in each MRI voxel. The relationship between NODDI and DTI metrics has been investigated in detail in previous works.^26^, ^27^ For instance, a decrease in DTI FA can be caused independently by a decrease in the density of axons or an increase in the orientation dispersion.

### Histological procedures

Optimized histological procedures^6^, ^32^ were followed.

The four samples were dehydrated and embedded in paraffin. Subsequently, 10 *μ*m‐thick sections were cut with a microtome from the surface exposed by the preliminary mid‐sagittal cut. The position of the sections within the sagittal MRI slices was inferred from the axial scans, accounting for tissue shrinkage.

For each case, serial histological sections mapped to two predefined MRI slices were stained for the following (two sections per MRI slice per staining, 200 *μ*m apart): Palmgren silver staining and phosphorylated and nonphosphorylated neurofilament immunostaining (neuronal elements); myelin proteolipid protein (PLP) immunohistochemistry (myelin); immunohistochemical labeling of ionized calcium‐binding adapter molecule 1 antigen (Iba1+) (microglia); glial fibrillary acidic protein (GFAP) immunohistochemistry (astrocytes).

### Optical imaging

High‐resolution digital images of the stained sections were acquired with an Aperio slide scanner (ScanScope AT Turbo) at a magnification of 400×. Data S2 shows examples of images from all specimens.

### Histological image processing

**Figure 1:**
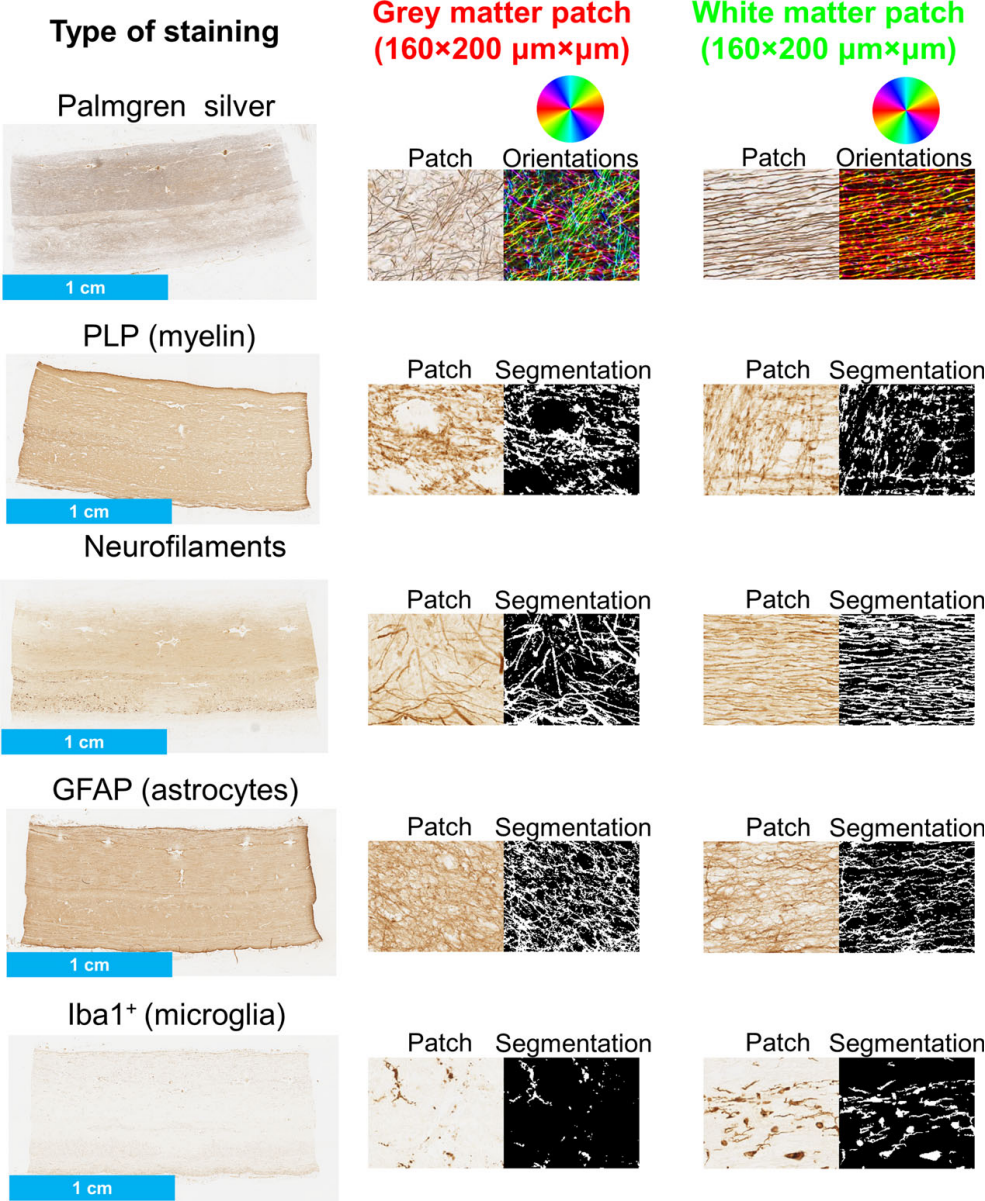
Examples of features in normal control samples from histological images. In the first column, an example of each staining from the upper lumbar control sample is shown. In the central and left columns, examples of raw image patches (160 × 200 *μ*m × *μ*m) from gray and white matter are pictured beside the result of the image processing. Specifically, image processing provided estimates of local neurite orientation (Palmgren silver staining) and segmentations of the stained material for the immunostains (PLP, neurofilaments, GFAP and Iba1+). Neurite orientations were used to evaluated patch‐wise orientation dispersion (metric CV), while the segmented areas to quantify patch‐wise staining fraction (MSF, NSF, ASF,* μ* GSF).

Histological images were processed in Matlab with the *StructureTensorToolbox* (http://github.com/fragrussu/StructureTensorToolbox) at a resolution of 1.008 × 1.008 *μ*m^2^. Processing enabled the calculation of the following features (Table 1) within patches matching the within‐slice MRI resolution (0.16 × 0.20 mm^2^): neurite orientation dispersion; fraction of material stained by each immunohistochemical labeling method (Fig. 1).

Neurite orientation dispersion was quantified by patch‐wise circular variance (CV) of neurite orientations (weighted‐Watson model^33^). Neurites were identified on the Palmgren images (4‐cluster *k‐means* segmentation^34^), and their orientation calculated with *structure tensor analysis* 33, 35 (spread of Gaussian kernels^33^: 1 *μ*m). Similarly to ODI, CV is an index ranging from 0 to 1, such that the variability of neurite orientations increases as CV increases.

The immunolabeled post mortem material was segmented with a 3‐cluster *k‐means* algorithm. Afterward, patch‐wise fractions of segmented material were calculated, obtaining four indices, ranging from 0 to 1 (Table 1): astrocyte staining fraction (ASF, from GFAP images); microglia staining fraction (*μ*GSF, from Iba1 images); myelin staining fraction (MSF, from PLP images); and neurofilament staining fraction (NSF, from neurofilament images).

### MRI‐histology co‐registration

Histological images and patch‐wise metrics were co‐registered in Matlab to the corresponding MRI slices with a landmark‐guided nonlinear approach,^36^ which maximized the Dice coefficient^37^ between the outlines of the specimens in MRI and histology.^38^ Landmark points were drawn on the mean *b* = 0 image and on the downsampled histological images.

### Region‐of‐interest outlining

Regional variation was assessed by studying different regions‐of‐interest (ROIs), drawn manually on the mean *b* = 0 images. Each ROI encompassed one particular tissue type: gray and white matter, for controls; nonlesional gray and nonlesional white matter, as well as lesions in gray and white matter, for multiple sclerosis cases. ROI‐wise medians of co‐registered NODDI and histological metrics were extracted (values from the two histological sections derived from each MRI slice were averaged).

### Statistical analysis

We hypothesized that NODDI metrics are associated with specific features of tissue microstructure, since NODDI aims to disentangle key morphological factors contributing to the diffusion‐weighted signal. We tested this hypothesis by evaluating the statistical associations between ROI‐wise median values of NODDI and histological indices, for a total of *N* = 48 ROIs (28 for the multiple sclerosis cases; 20 for the controls), similar in number to other studies.^17^, ^20^

Firstly, we calculated Pearson's correlation coefficients between NODDI and histological metrics in multiple sclerosis and control cases in turn (significance level: *P* < 0.05). Secondly, we fitted univariate multivariable linear regression models to quantify the sensitivity of each NODDI metric toward each histological index. We chose MRI indices as the dependent variables (in turn), since one would use MRI as a noninvasive probe of the underlying, histological features. Histological indices were considered as the predictors of the MRI metrics, as neuropathological analyses capture the micro‐architectural substrates of the MR signal. We fitted five linear regression models (Table 2) in controls and multiple sclerosis cases in turn as follows.

1. Model number 1 is the most general. It employs all histological features as predictors.
2. Model number 2 considers only metrics related to neurons (CV, MSF, NSF) as predictors of each NODDI index. From model number 2, two more models were obtained: model 3 and 4.
3. Model number 3 focusses on the joint dependence of NODDI indices on density and dispersion of neuronal elements (metrics NSF, CV). These two are key factors contributing to the diffusion MRI signal,^26^ and it is relevant to determine which is more important to explain the variability in NODDI metrics.
4. Model number 4 was formulated to study specifically demyelination and axonal/neuronal loss. The model employs myelin and neurofilament density as predictors, and allows the quantification of the sensitivity of each MRI metric to each of these two.
5. Model number 5 evaluates the association between NODDI metrics and features of the extra‐neuronal space (i.e., glial fractions ASF and *μ*GSF). Comparing the quality of fit of this model to that of model 2 assesses whether MRI metrics are more strongly related to neuronal features or to features of the extra‐neuronal space.

**Table 2:** Linear regression models fitted in this study

Model number	Equation
1	*m* = *β*_0_ + *β*_1_ CV + *β*_2_ MSF + *β*_3_ NSF + *β*_4_ ASF + *β*_5_ *μ*GSF
2	*m* = *β*_0_ + *β*_1_ CV + *β*_2_ MSF + *β*_3_ NSF
3	*m* = *β*_0_ + *β*_1_ CV + *β*_2_ NSF
4	*m* = *β*_0_ + *β*_1_ MSF + *β*_2_ NSF
5	*m* = *β*_0_ + *β*_1_ ASF + *β*_2_ *μ*GSF *m* stands for the generic NODDI (IVF, NDI, ODI) or DTI (FA, AD, RD, MD) metric.

Linear regressions were performed in R (https://www.r-project.org/), obtaining standardized *β*‐coefficients (association with the histological metrics; significance level *P* < 0.05) and adjusted coefficients of determination (quality of fit). The correlations and the regressions were also evaluated for standard DTI FA, AD, RD, and MD for comparison.

## Results

The key results of this paper are that:

1. Both MRI (ODI) and histology (CV) reveal a trend of lower neurite orientation dispersion in multiple sclerosis demyelinated lesions, indicative of reduced geometrical complexity of the neurite architecture
2. NODDI is sensitive and specific to the underlying orientation dispersion as measured by histology in post mortem spinal cord tissue from multiple sclerosis and nonneurological controls (significant association between MRI and histological dispersion: *P* < 0.001).

### Maps of quantitative metrics from NODDI and histology

**Figure 2:**
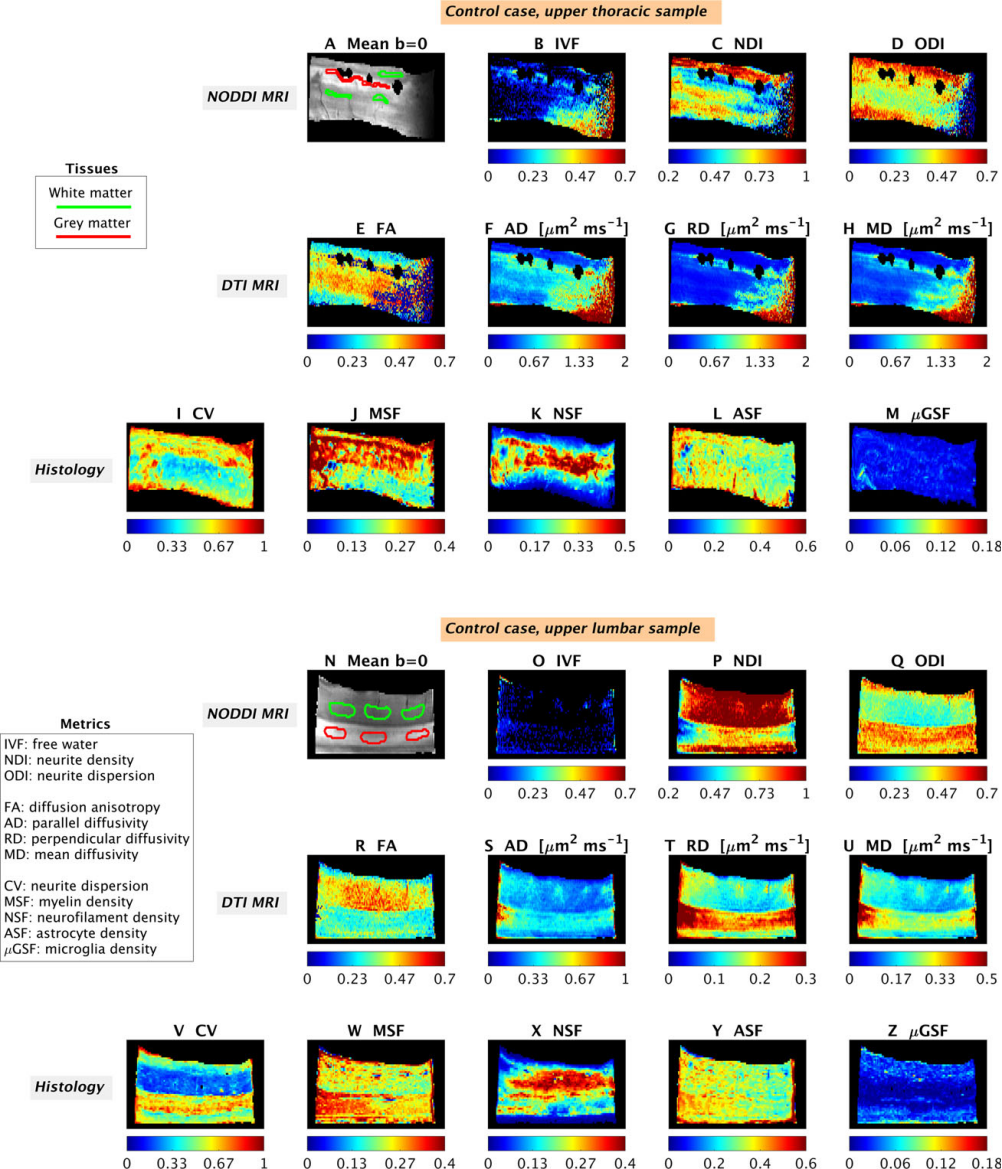
Examples of quantitative maps from NODDI and histology in the control cases. Top: upper thoracic sample; bottom: upper lumbar sample. From MRI: mean *b* = 0 image with manual regions‐of‐interest (A and N); IVF (amount of free water, B and O); NDI (neurite density index, C and P); ODI (dispersion of neurite orientations, D and Q); FA (diffusion anisotropy, E and R); AD (diffusivity along principal tensor direction, F and S); RD (diffusivity across principal tensor direction, G and T); MD (mean rate of diffusion, H and U). From histology: CV (dispersion of neurite orientations: I and V); MSF (amount of myelin: J and W); NSF (amount of neurofilaments: K and X); ASF (amount of astrocytes: L and Y); *μ* GSF (amount of microglia: M and Z). The black areas especially visible in A (upper thoracic control) are artifacts due to distortions caused by the proximity of residual air bubbles, which were masked out when visualizing the maps (B to H).

**Figure 3:**
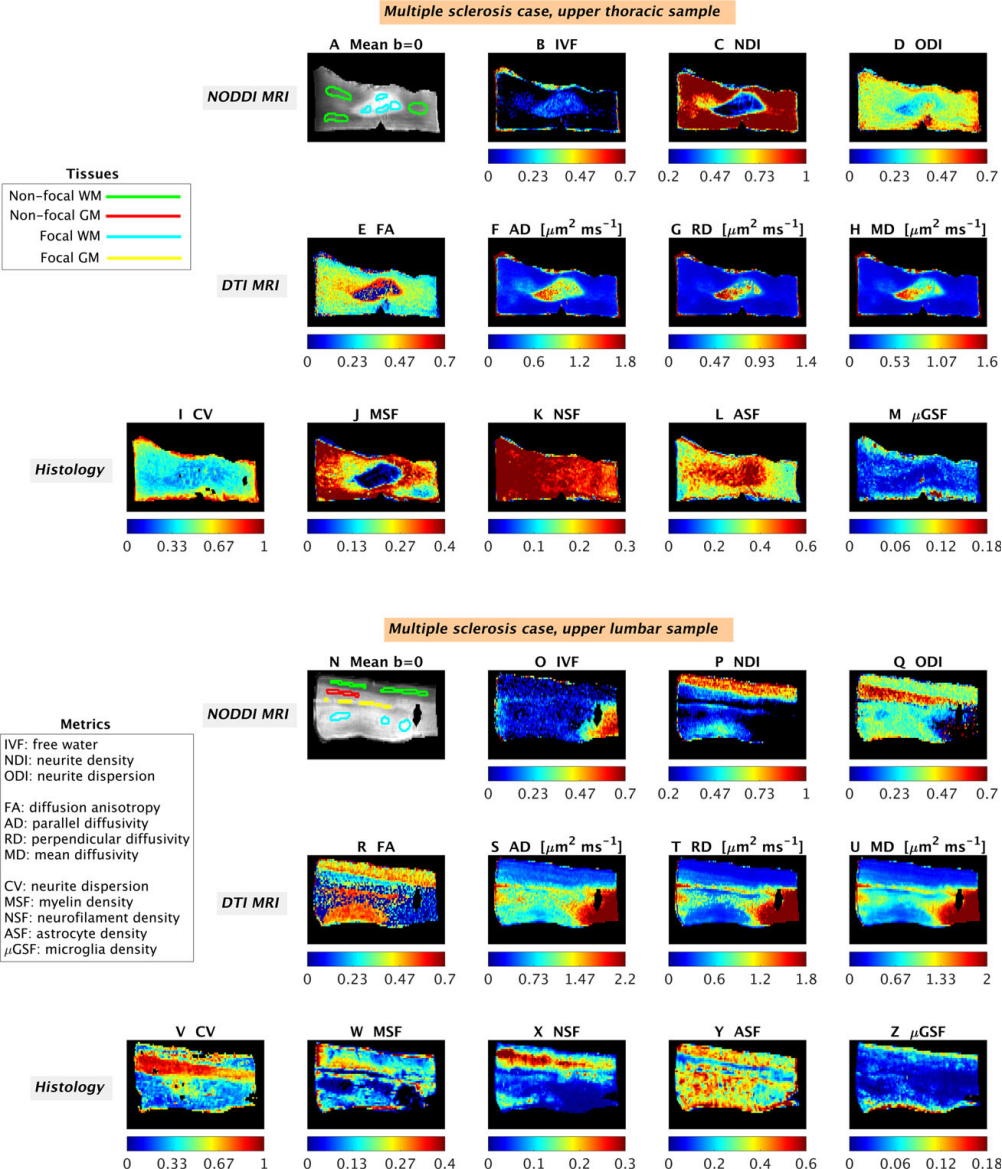
Examples of quantitative maps from NODDI and histology in the multiple sclerosis cases. Top: upper thoracic sample; bottom: upper lumbar sample. The meaning of the metrics is that same as figure 2, and it is summarized in figure 2 caption. From MRI: mean *b* = 0 image with manual regions‐of‐interest (A and N); IVF (B and O); NDI (C and P); ODI (D and Q); FA (E and R); AD (F and S); RD (G and T); MD (H and U). From histology: CV (I and V); MSF (J and W); NSF (K and X); ASF (L and Y); *μ* GSF (M and Z). The black areas especially visible in N (upper lumbar multiple sclerosis specimen) are artifacts due to distortions caused by the proximity of residual air bubbles, which were masked out when visualizing the maps (O to U).

Quantitative MRI and histological metrics and examples of ROIs are illustrated for control (Fig. 2) and multiple sclerosis cases (Fig. 3).

#### Nonneurological control observations

Histological analysis of the upper thoracic and lumbar cords of the two control cases did not show evidence of gray or white matter demyelination.

In both specimens, NODDI IVF is similarly low in both gray and white matter. In contrast, NDI is higher in white compared to gray matter whereas the converse is true for ODI. Histological staining fractions (MSF, NSF, ASF, and *μ*GSF) show little contrast between gray and white matter. CV is higher in gray matter compared to white matter. DTI metrics show known trends,^36^ such as higher diffusion anisotropy in white as compared with gray matter.

#### Multiple sclerosis observations: thoracic specimen

In the upper thoracic cord specimen, we observe a solitary chronic inactive demyelinated white matter lesion with a paucity of microglial inflammation, relative axonal preservation, and associated astrogliosis. These observations are captured in the histological metrics wherein MSF is decreased (myelin loss), *μ*GSF is decreased (microglia reduction), ASF is increased (astrogliosis), and NSF is unchanged (neurofilament preservation) in the focal white matter lesion compared to nonlesional white matter areas. CV is reduced within the focal demyelinated white matter lesion compared to nonlesional areas reflecting reduced variability in axonal orientations within the lesion. NODDI MRI maps demonstrate increased IVF and decreased NDI and ODI in this lesion compared to nonlesional areas. DTI metrics show variations in FA (with both areas of increase and of decrease), and increased AD, RD, and MD.

In nonlesional areas, differences between gray and white matter are observed with increased CV (histology), increased ODI (MRI), and decreased NDI (MRI) in gray compared to white matter as seen in controls. DTI metrics also show similar contrasts as those of controls in nonlesional areas (quantitative maps not shown).

#### Multiple sclerosis observations: lumbar specimen

In the upper lumbar cord specimen, separate demyelinated lesions in each of gray and white matter, are seen. All lesions are chronic inactive demonstrating loss of myelin and an absence of microglial inflammation. Dissimilar to the upper thoracic cord specimen, the gray and white lesions in the lumbar cord specimen show a significant reduction in neurofilament density (reflecting axonal loss) and an absence of pronounced astrogliosis. Quantitative histological maps reflect these features with a decrease in MSF (myelin loss), NSF (axonal loss), and *μ*GSF (microglia reduction) and no change in ASF (astrogliosis) in lesional compared to nonlesional areas (Fig. 3). Despite the differences between thoracic and lumbar cord specimens, CV is reduced within the lesional compared to nonlesional areas once again reflecting reduced neurite orientation variability within the lesion. NODDI maps demonstrate a trend towards a decrease (NDI and ODI) or increase (IVF) in focal lesions as compared to surrounding nonlesional tissue. DTI maps show changes in FA (decreased) and AD, RD, and MD (all increased) in lesional areas, as compared to nonlesional tissue.

In nonlesional areas, differences between gray and white matter are observed in the quantitative histological index CV, which decreases in gray compared to white matter as is seen in controls. In the areas sampled, NODDI and DTI maps (MRI) show that (1) IVF is uniformly low in nonlesional gray and white matter; (2) NDI is higher in nonlesional white compared to nonlesional gray matter and; (3) ODI is higher in nonlesional gray than in nonlesional white matter; (4) FA is lower in nonlesional gray than nonlesional white matter; (5) diffusivities (AD, RD, and MD) show contrast between nonlesional gray and nonlesional white matter, with the former often exhibiting higher values of these metrics. Histological maps demonstrate increased ASF (astrocyte density) in white compared to gray matter. In contrast, measures of MSF (myelin), NSF (neurofilament), and *μ*GSF (microglia) are similar between white and gray matter.

### Scatter plots of voxel‐wise values

**Figure 4:**
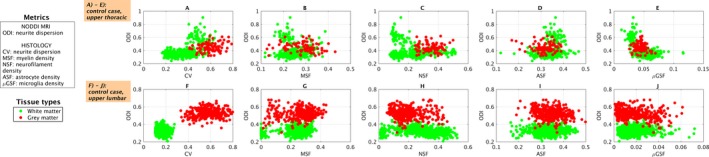
Scatter plots of NODDI orientation dispersion (ODI) and histological metrics (CV in A and F; MSF in B and G; NSF in C and H; ASF in D and I; *μ* GSF in E and J). The plots are color‐coded according to tissue type (tissue types also shown in Fig 2). Top: upper thoracic control sample (A to E). Bottom: upper lumbar control sample (F to J). Green dots correspond to voxels from white matter, red from gray matter.

**Figure 5:**
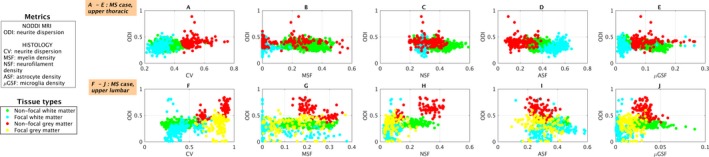
Scatter plots of NODDI orientation dispersion (ODI) and histological metrics (CV in A and F; MSF in B and G; NSF in C and H; ASF in D and I; *μ* GSF in E and J). The plots are color‐coded according to tissue type (tissue types also shown in Fig 3). Top: upper thoracic multiple sclerosis sample (A to E). Bottom: upper lumbar multiple sclerosis sample (F to J). Green dots correspond to voxels from nonlesional white matter; light blue from lesional white matter; red from nonlesional gray matter; yellow from lesional gray matter (note that no lesional gray matter was identified in the upper thoracic sample).

We observe regional variation in NODDI ODI and of histological metrics as illustrated by voxel‐wise scatter plots in control (Fig. 4) and multiple sclerosis cases (Fig. 5). The plots show patterns that are in line with those reported in the previous section: a trend towards reduced ODI and CV of lesional gray/white matter as compared by nonlesional tissue of the same type; higher orientation dispersion in the gray matter than in the white matter of control cases. Histological CV replicates well the contrast in both controls and multiple sclerosis cases. In the upper thoracic case, notably ASF values shows changes in their distributions between lesional and nonlesional white matter that mirror well those of ODI, although with reversed contrast.

### Statistical analysis

#### Pearson's correlation

**Figure 6:**
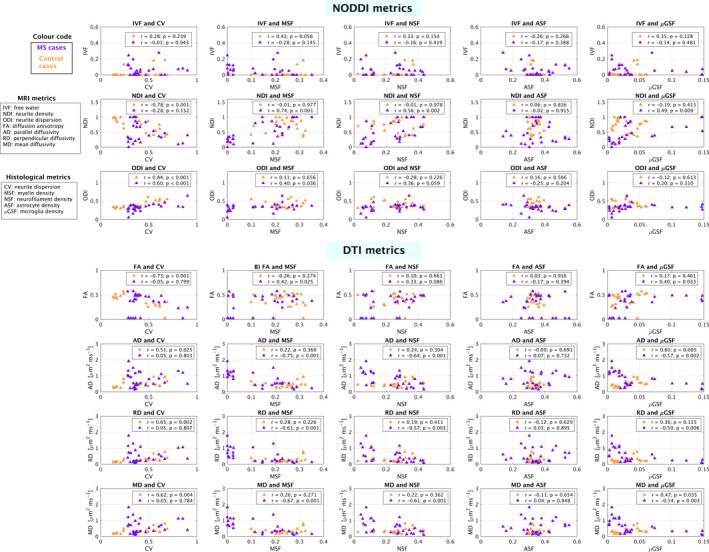
Summary of pair‐wise associations between MRI metrics (NODDI and DTI) and histological indices. Associations are reported as Pearson's correlation coefficients (indicated as *r*) and were evaluated separately for controls (orange) and multiple sclerosis cases (violet). Data points represent median values within several regions‐of‐interest, outlined on the mean *b* = 0 MRI images after co‐registration MRI‐histology.

ROI‐wise scatter plots and Pearson's correlation coefficients *r* (Fig. 6) reveal a strong association between ODI and its histological counterpart CV, observed consistently in control and multiple sclerosis cases.

We find a strong positive correlation between ODI and CV in both control (*r* = 0.84; *P* < 0.001) and multiple sclerosis cases (*r* = 0.60; *P* = 0.001), as well as a weaker positive correlation with MSF in multiple sclerosis (*r* = 0.40; *P* = 0.036). A negative correlation is seen between NDI and CV (*r* = −0.78; *P* < 0.001) in controls, as well as a strong positive correlation with MSF (*r* = 0.74; *P* < 0.001), NSF (*r* = 0.56; *P* = 0.002) and a weaker correlation with *μ*GSF (*r* = 0.49; *P* = 0.009) in multiple sclerosis cases. No significant correlations are observed for NODDI IVF.

Regarding DTI metrics, a negative correlation is seen between FA and CV (*r* = −0.73; *P* < 0.001) in controls, as well as a positive correlation between FA and MSF (*r* = 0.42; *P* = 0.025) and FA and *μ*GSF (*r* = 0.40; *P* = 0.033) in multiple sclerosis. AD shows a positive correlation with CV (*r* = 0.51; *P* = 0.025) and *μ*GSF (*r* = 0.60; *P* = 0.005) in controls, and a negative correlation with MSF (*r* = −0.75; *P* < 0.001), NSF (*r* = −0.64; *P* < 0.001) in multiple sclerosis. RD is positively associated to CV (*r* = 0.65; *P* = 0.002) in controls, and negatively associated to MSF (*r* = −0.61; *P* < 0.001), NSF (*r* = −0.57; *P* = 0.001) and *μ*GSF (*r* = −0.50; *P* = 0.006) in multiple sclerosis. Lastly, MD shows a positive correlation with CV (*r* = 0.62; *P* = 0.004) and *μ*GSF (*r* = 0.47; *P* = 0.035) in controls, and with MSF (*r* = −0.67; *P* < 0.001), NSF (*r* = −0.61; *P* < 0.001) and *μ*GSF (*r* = −0.54; *P* = 0.003) in multiple sclerosis.

#### Linear regression

Significant associations from linear regression models are listed below (as standardized beta coefficients; full results available in Data S3). Overall, the association between ODI and CV, in both controls and multiple sclerosis cases, is the strongest and most significant and consistent among all detected associations.

1. *Model number 1*: in control cases, NDI is negatively associated with CV (*β* = −0.785, *P* = 0.0004) and ODI is positively associated with CV (*β* = 0.860, *P* < 10^−5^). FA is negatively associated with CV (*β* = −0.763, *P* = 0.002) while AD, RD, and MD are positively associated with CV (*β* = 0.459, *P* = 0.019 for AD; *β* = 0.638, *P* = 0.003 for RD; *β* = 0.590, *P* = 0.004 for MD). AD is also associated with *μ*GSF (*β* = 0.529, *P* = 0.011). In multiple sclerosis cases, NDI is positively associated with MSF (*β* = 0.677, *P* = 0.0002). ODI is positively associated with CV (*β* = 0.711, *P* < 10^−4^), and, to lesser extent, with NSF (*β* = 0.521, *P* = 0.013). DTI metrics AD, RD and MD show a negative association with MSF (*β* = −0.628, *P* = 0.0002 for AD; *β* = −0.496, *P* = 0.013 for RD; *β* = −0.544, *P* = 0.004 for MD).
2. *Model number 2*: in controls, NDI and ODI show, respectively a negative and positive association with CV (NDI: *β* = −0.789, *P* = 0.0002; ODI: *β* = 0.838, *P* < 10^−5^); FA shows a negative association with CV (*β* = −0.708; *P* = 0.001); AD (*β* = 0.508; *P* = 0.026), RD (*β* = 0.645; *P* = 0.002) and MD (*β* = 0.615; *P* = 0.005) all show a positive association with CV. In multiple sclerosis cases, NDI is negatively associated with CV (*β* = −0.258, *P* = 0.043) and positively associated with MSF (*β* = 0.647, *P* < 10^−4^); ODI shows a positive association with NSF (*β* = 0.403, *P* = 0.009), weaker than that with CV (*β* = 0.673, *P* < 10^−4^). DTI metrics AD, RD and MD all show negative associations with MSF and NSF (AD: *β* = −0.578, *P* = 0.0002 with MSF and *β* = −0.385, *P* = 0.008 with NSF; RD: *β* = −0.446, *P* = 0.012 with MSF and *β* = −0.380, *P* = 0.0317 with NSF; MD: *β* = −0.493, *P* = 0.003 with MSF and *β* = −0.397, *P* = 0.016 with NSF).
3. *Model number 3*: in control cases, NDI is negatively associated with CV (*β* = −0.760, *P* < 0.0002) and ODI is positively associated with CV (*β* = 0.830, *P* < 10^−5^). DTI metric FA is negatively associated with CV (*β* = −0.733, *P =* 0.0003), while DTI AD (*β* = 0.527, *P* = 0.017), RD (*β* = 0.673, *P* = 0.001) and MD (*β* = 0.640, *P* = 0.002) are positively associated with CV. In multiple sclerosis cases, NDI is positively associated with NSF (*β* = 0.521, *P* = 0.004) and ODI is positively associated with CV (*β* = 0.697, *P* < 10^−4^) and, to lesser extent, with NSF (*β* = 0.494, *P* = 0.0008). DTI AD (*β* = −0.652, *P* = 0.0003), RD (*β* = −0.586, *P* = 0.002) and MD (*β* = −0.625, *P* = 0.001) are all negatively associated with NSF.
4. *Model number 4*: no significant associations between NODDI and DTI metrics and histological NSF and MSF are detected in controls. In multiple sclerosis cases, NDI is positively associated with both MSF (*β* = 0.610; *P* = 0.0002) and NSF (*β* = 0.288; *P* = 0.050). AD, RD and MD are all negatively associated to MSF (AD: *β* = −0.579; *P* = 0.0001; RD: *β* = −0.447; *P* = 0.009; MD: *β* = − 0.494; *P* = 0.002) and NSF (AD: *β* = −0.384; *P* = 0.006; RD: *β* = −0.378, *P* = 0.025; MD: *β* = − 0.396; *P* = 0.012).
5. *Model number 5*: no significant associations between NODDI metrics and glial fractions ASF and *μ*GSF are detected in controls, while DTI AD (*β* = 0.632; *P* = 0.004) and DTI MD (*β* = 0.501; *P* = 0.030) show a positive association with *μ*GSF. In multiple sclerosis cases, NDI is positively associated with *μ*GSF (*β* = 0.508; *P* = 0.009), while AD (*β* = −0.581; *P* = 0.002), RD (*β* = −0.527; *P* = 0.006) and MD (*β* = −0.558; *P* = 0.003) are negatively associated to *μ*GSF.

Regarding the adjusted coefficients of determinations, the following is observed.

NODDI metrics: for models 1 to 3, higher values of the adjusted coefficients of determination for ODI and NDI as compared to IVF are obtained (maximum/minimum values: 0.740/0.566 for ODI; 0.630/0.286 for NDI; 0.332/−0.051 for IVF). For model 4, very low values of this coefficient are observed for ODI and IVF (control cases: −0.010 for ODI, 0.194 for IVF; multiple sclerosis cases: 0.137 for ODI, 0.008 for IVF) and NDI in control cases (−0.118), while a higher value is obtained for NDI in multiple sclerosis (0.577). Finally, model number 5 provides low values of the adjusted coefficient of determination for both control and multiple sclerosis cases and all NODDI metrics (maximum value for NDI in multiple sclerosis cases of 0.184; minimum for NDI in control cases of −0.068).

DTI metrics: the adjusted coefficients of determination are of similar magnitude as those of NODDI indices (full details in Data S4).

## Discussion

### Key findings

Our study tests the hypothesis that the variability of neurite orientations is a biomarker of multiple sclerosis pathology, and is motivated by recent findings demonstrating that neurite morphology is a substrate of neural function^23^, ^24^ and is affected by multiple sclerosis.^25^

Through the combination of state‐of‐the‐art histology^33^ and post mortem NODDI^26^, ^27^ MRI, we show for the first time that neurite orientation dispersion is a marker of microstructural pathology, as it detects trends of reduced geometrical complexity of neurite architecture within multiple sclerosis lesions. In so doing, we report a heretofore undescribed layer of complexity of multiple sclerosis pathology. Also, we provide unequivocal evidence that NODDI dispersion indices are histologically meaningful, demonstrating their clinical viability since NODDI can be set up in clinical systems.^26^, ^27^

### Patterns of quantitative metrics

We present quantitative maps (Table 1) from NODDI (IVF, NDI, and ODI), DTI (FA, AD, RD, and MD) and histology (CV, MSF, NSF, ASF, *μ*GSF) at the same resolution and from the same locations (Figs. 2 and 3). We also illustrate regional variation in orientation dispersion and all histological metrics with scatter plots (Figs. 4 and 5), which confirm observations from visual inspection.

In controls, NODDI NDI and ODI show contrast between gray and white matter, in line with recent in vivo results.^27^ NDI is higher in white matter than in gray matter, while the opposite holds for ODI. The histology‐derived index of orientation dispersion, CV, confirms the patterns exhibited by ODI: dispersion is higher in gray than white matter, due to the established higher variability in dendrite orientations compared to axons. However, the histology‐derived index of neuronal element density, NSF, does not exhibit such a clear contrast between gray and white matter. Several factors may explain this observation. Neurofilament immunostaining demonstrates neuronal cell bodies, potentially reducing the gray/white matter contrast of NSF, while differences in myelination between dendrites and axons may increase the gray/white matter contrast of NDI. Regarding DTI metrics, known contrasts are observed between controls’ gray and white matter.^27^, ^36^

Multiple sclerosis specimens provide a unique opportunity to evaluate several cytoarchitectural compositions, including gray versus white matter in both nonlesional and lesional (i.e., demyelinated) states. In nonlesional tissue, NODDI metrics in gray and white matter follow a similar pattern as that described in controls, as well as those from DTI. In contrast, lesional matter shows hypointense NDI and ODI maps and hyperintense IVF relative to nonlesional areas, in line with recent findings in vivo.^39^ DTI metrics highlight focal pathology as well, with increased diffusivity and changes in FA, similarly to what has been previously reported.^36^ Our results suggest that quantitative diffusion MRI metrics are well positioned to detect areas of focal pathology in the spinal cord.

We provide novel insight into the histological substrate of NODDI metrics. Specifically, we show that ODI corresponds well with histologically derived CV (representing neurite orientation dispersion) and that NDI can differ from histologically derived NSF (i.e., neurofilament staining fraction). The implications of these findings will be discussed below.

NODDI ODI detection of reduced orientation dispersion in lesions confirmed by histological CV is a key finding of this work. This observation suggests that within areas of focal demyelination in the spinal cord, neurites can have reduced orientation variability. In white matter, this may reflect reduced collateral branching or morphological alterations of individual axons.^40^ In gray matter, reduced orientation dispersion may imply reduction in the complexity of dendritic arborisations. Reduced orientation dispersion is entirely consistent with recent research in multiple sclerosis, which has reported: reduced dendrite branching in cortical gray matter^25^; reduced diffusion‐MRI cortical complexity^41^; increased cortical lesion fractional anisotropy.^42^ Our work highlights how the complexity of neurite composition can be altered not only in the multiple sclerosis cortex, but also in the functionally relevant spinal cord. Notably, DTI FA also highlights changes within certain parts of the focal lesion in the upper thoracic case are in line with reduced dispersion (i.e., increased FA). However, the upper lumbar multiple sclerosis case highlights how in other cases FA is confounded by demyelination, as it decreases when NODDI ODI and histological CV also decrease (note that a decrease in dispersion would imply an increase in FA, for fixed neurite density^27^). Hence, the strong dependence on other factors other than neurite dispersion makes DTI metrics a poorer tool than NODDI ODI to measure changes of neurite geometry complexity due to multiple sclerosis pathology.

The relationship within focal lesions between NSF (histological neuro‐axonal density) and NDI (MRI‐derived neurite density) is complex. While NDI always decreases within lesions, NSF does not necessarily behave similarly, highlighting variable degrees of axonal loss in different lesions (Fig. 2). In contrast, NDI always drops dramatically within lesions, similar to myelin density MSF. This finding is not surprising as NDI is a surrogate index equivalent to NSF/(1–MSF)^26^, ^43^ (post hoc analysis confirms a correlation between these two measures). Although altered exchange of water between intra/extra‐axonal space (i.e., permeability)^40^, ^44^ may have contributed to the observed patterns of NDI, it is expected that variations of myelin density would also contribute. Myelin water is virtually invisible in diffusion MRI due to very short T2.^45^ When myelin is lost, the local T2 increases,^46^ effectively increasing the amount of MRI‐visible water and causing NDI to decrease.^26^

All in all, our work highlights how focal multiple sclerosis pathology can be characterized at a new level, via analysis of heretofore unexplored features such as neurite orientation dispersion. While innovative, our results are certainly preliminary and should be interpreted with care. Further research is required both ex vivo and in vivo to substantiate our findings and study systematically the prognostic value of dispersion measurements. Larger sample sizes will be required in the future to enable a better characterization of the spatial distribution of dispersion changes due to multiple sclerosis pathology. For instance, a preliminary investigation reported as supplementary material (Data S4) highlights how MRI metrics may be able to detect pathology that extends from focal lesions in the peri‐plaque area. Yet, evidence from a larger number of ex vivo specimens is required to confirm these changes histopathologically, due to the inherent variability in the labeling efficiency at very short spatial scales.

### Statistical analysis

The main finding highlighted by correlation and linear regression analyses is that NODDI ODI is sensitive and highly specific to histologically derived neurite orientation dispersion in the presence of multiple sclerosis‐related pathology. Furthermore, NODDI metrics are specific to neurons and are not influenced by features of the extra‐neuronal space, such as density of glial cells. Importantly, NODDI NDI offers sensitivity to the local density of axon/dendrites but is also strongly influenced by variations of myelination, limiting its interpretability without the support of myelin mapping techniques. Further, markers of histology do not relate to IVF, which is not surprising given that IVF is an indicator of free water and our histological samples are dehydrated in their preparation.

In controls, the histological orientation dispersion CV is the main explanatory factor of NODDI NDI and ODI, resulting from its high gray/white matter contrast. In multiple sclerosis, the statistical analysis reveals several interesting observations. ODI shows a strong, positive association with its direct histological counterpart CV. ODI shows only a weak correlation with MSF in multiple sclerosis cases (not confirmed by linear regression), likely due to the similar contrast of the two between lesional and nonlesional tissue. Additionally, ODI also shows a weaker, positive association with NSF. The association of ODI‐NSF in multiple sclerosis may result from the similar contrast between the two maps due to the presence of lesions, or may reflect a concomitant effect of the disease, which may alter the number of neurites while also changing their underlying geometry. Finally, linear regression models confirm that NODDI NDI offers sensitivity to the density of neuronal elements (positive association with NDI‐NSF in model 3), but is also strongly influenced by the local amount of myelin.

The statistical analysis also shows important insight into DTI metrics. Both correlation and linear regression analyses confirm once more that DTI is very sensitive to multiple sclerosis pathology, as shown by strong changes of FA, AD, RD, and MD noticeable on simple visual inspection.^36^ However, DTI metrics appear as surrogate biomarkers of multiple sclerosis pathology with relatively poor specificity, as they are jointly influenced by demyelination, neuroaxonal loss and even changes in the glial component. Of note, no DTI metrics show association with histology‐derived dispersion (CV), unlike NODDI ODI. This latter finding highlights how biophysical models such as NODDI have the potential of increasing the specificity of imaging biomarkers towards unique morphological features of neuronal tissue, and may be able to open new windows for the characterization of pathology at the neurite scale.

### Confounding factors

Care was taken to consider the influence of confounding factors. We verified that histological section thickness did not vary between lesional and nonlesional tissue (data not shown) and excluded areas of nonspecific staining with a combination of manual and automatic segmentation to calculate CV, given their potential to bias this metric.^33^

### Limitations

We acknowledge a number of limitations.

NODDI metrics were obtained with a nonclinical MRI protocol and from ex vivo fixed tissue. Fixation can alter tissue microstructure^47^, ^48^; therefore, NODDI metrics as shown here may not be fully representative of their in vivo counterparts. Nevertheless, our results agree well with preliminary findings in vivo*,* 39, 49 providing confidence that the conclusions of this study will most likely hold true in clinical scenarios. A recent study has highlighted changes of NODDI metrics in the spinal cord in vivo, although at a different level and on a different subtype of disease.^29^ Similar works highlight that for the in vivo application of the NODDI technique signal‐to‐noise ratio is a key factor, and careful optimization of the sequence parameters is required in order to obtain reliable microstructural indices.

Also, we obtained 2D histological indices from tissue sections whose thickness was considerably smaller than that of the MRI slices (10 *μ*m vs. 800 *μ*m), to achieve a satisfactory signal‐to‐noise ratio in MRI. To account for this mismatch, we derived two different histological sections per MRI slice (200 *μ*m apart), and averaged the histological indices for analysis. Nonetheless, not all the tissue that contributed to the MRI signal was sampled, possibly explaining some of the differences between MRI and histology.

Another limitation relates to the Palmgren silver method, which impregnates neurites in black and surrounding nonneural tissue in lighter shades of brown. Most of these structures were removed with manual and automatic segmentation, but it is possible that some may have remained. Nevertheless, tests demonstrated that the inclusion of nonspecific staining did not change the between‐tissue contrast (data not shown).

Furthermore, we also acknowledge that the number of specimens employed in this study (i.e., four) is limited, although results are compelling and exciting. Therefore, further research is required to confirm the changes in neurite dispersion due to multiple sclerosis pathology reported here. Regarding the MRI‐histopathology correlations, it should be noted that each specimens offers the possibility of investigating several different types of cytoarchitecture, which were probed by drawing ROIs in all types of tissue. Practically, this led to the construction of a data set of 48 data points, whose size suffices to fit the regression models and evaluate correlations (other high‐impact studies on MRI‐histopathology correlation relied on the same amount of data^20^ or even less^17^).

Regarding the NSF maps, neurofilament antigenicity can be negatively affected by formalin fixation,^6^ which may be at least in part responsible for some of the heterogeneity shown in our neurofilament immunolabeling. Our analysis accounted for spatial trends of intensity, but residual nonuniformities may have led to the underestimation of the association NDI‐NSF.

In this study we only considered a limited number of metrics (NODDI and DTI) derived from MRI. It is possible that other indices, either from other diffusion techniques^19^, ^43^ or from other MRI modalities (relaxometry, magnetization transfer techniques, susceptibility imaging or others) may have shown even stronger histopathological correlations. The main focus of this work was neurite orientation dispersion. Our particular MRI implementation, based on one dispersion mapping technique among many (NODDI), aimed to show that mapping this often neglected feature of neuronal microstructure has strong clinical potential.

Finally, we point out that this work shows the potential utility of NODDI metrics, but also highlights some caveats related to their interpretation. We stress that NODDI metrics should always be interpreted with care: NODDI indices are designed to measure geometrical features of neurite morphology, but in practice they can be influenced by other factors, as for example myelin for the case of NDI.

### Future directions

Future validation of our preliminary findings could include: analysis of tissue from areas beyond the spinal cord; extension of the histology to the third dimension^50^, ^51^; further confirmation from in vivo data; characterization of more complex morphological features of glial cells^52^; more accurate diffusion MRI signal modeling^43^, ^53^; analysis of other quantitative MRI metrics (such as those from relaxometry, magnetization transfer techniques or susceptibility imaging).

## Conclusions

Our study identifies a novel sensitive and specific biomarker of microstructural pathology in the multiple sclerosis spinal cord: neurite orientation dispersion. Moreover, our work highlights how a clinically viable quantitative MRI method, NODDI, provides a histologically meaningful measure of neurite dispersion that can be used to unravel previously undetected layers of complexity of multiple sclerosis pathology in the spinal cord. The in vivo application of neurite orientation dispersion mapping has the potential to not only cast light onto pathophysiological processes relevant to multiple sclerosis, but also to provide a clinically sensitive outcome measure in clinical trial and practice settings for prognosis and monitoring treatment response.

## Author Contributions

Concept and study design: F.G., T.S., C.T., J.N., H.Z., D.C.A., G.C.D., C.G.W.K. Acquisition and analysis of data: F.G., T.S., C.T., R.L.Y, M.T., A.I., M.C.Y, D.C.A., G.C.D., C.G.W.K. Drafting manuscript and figures: F.G., T.S., C.T., R.L.Y., M.T., A.I., M.C.Y., J.N., H.Z., D.C.A., G.C.D., C.G.W.K. F.G., and T.S. are joint first authors. G.C.D. and C.G.W.K. are joint senior authors.

## Conflicts of Interest

T.S. is an employee of Philips UK. G.C.D. has received: travel expenses from Bay Schering, Biogen Idec, Genzyme, Merck Serono, Novartis; honoraria as an invited speaker for Bayer Schering; research funding from Merck‐Serono. C.T. has received honoraria and support for travelling from Bayer‐Schering, Teva, Merck‐Serono and Serono Foundation, Biogen, Sanofi‐Aventis, Novartis, and Ismar Healthcare.

## Supporting information


**Data S1**. Tissue samples and MRI‐histology pipeline.

**Data S2**. Examples of histological images.

**Data S3**. Full results from linear regression models.

**Data S4**. Quantitative metrics in white matter peri‐plaque areas.
