# Supplementary material for: Neurite dispersion: a new marker of multiple sclerosis spinal cord pathology?
Source: Ann Clin Transl Neurol. 2017 Aug 15;4(9):663–79. doi: 10.1002/acn3.445 (PMC5590517; doi:10.1002/acn3.445)
Supplement: Supplementary file 1 — Data S1. Tissue samples and MRI‐histology pipeline. [file ACN3-4-663-s001.pdf]

# Supplementary material 1: samples and MRI-histology pipeline

In this supplementary material, we provide additional information about the spinal cord specimens and decribe in detail the procedure that was followed to obtain MRI and histology data.

## Spinal cord tissue specimens

Supplementary table 1. 1 provides information regarding the four spinal cord specimens included in the study. Supplementary table 1. 2 provides further details about the two multiple sclerosis (MS) cases.

| Case | Sex | Age [years] | Cause of decease | Decease-to-fixation [hours] | Cord level | Length [cm] |
|---|---|---|---|---|---|---|
| Control | M | 66 | Cardiac arrest | $< 48$ | Upper thoracic | 3.1 |
| Control | F | 67 | Infective exacerbation of severe end-stage COPD | $< 48$ | Upper lumbar | 2.1 |
| MS | M | 75 | Asphyxiation, secondary to MS | $< 48$ | Upper thoracic | 3.3 |
| MS | F | 67 | Pneumonia, secondary to MS | 23 | Upper lumbar | 2.4 |

Supplementary table 1. 1: information regarding the four spinal cord specimens included in the study. COPD stands for *chronic obstructive pulmonary disease*. MS stands for *multiple sclerosis*.

| Sex | Age at decease [years] | Cause of decease | Cord level | Length [cm] | Disease subtype | Disease duration [years] | EDSS before death |
|---|---|---|---|---|---|---|---|
| M | 75 | Asphyxiation, secondary to MS | Upper thoracic | 3.3 | PPMS | 21 | 6.5 |
| F | 67 | Pneumonia, secondary to MS | Upper lumbar | 2.4 | SPMS | 33 | 9.5 |

Supplementary table 1. 2: additional details about the specimens of spinal cord tissue obtained from the MS cases. PPMS stands for *primary progressive multiple sclerosis*; SPMS for *secondary progressive multiple sclerosis*.

## MRI-histology pipeline

We designed a strategy to determine the radiographic position of the histological material in the sagittal, diffusion-weighted MRI images. It consists of: i) sectioning mid-sagittally the spinal cord specimens prior to MRI; ii) acquiring the sagittal MRI slices parallel to surface exposed by the cut; iii) deriving the histological material also in parallel from the exposed midsagittal surface. The steps of the pipeline are described in detail below. A block diagram of the pipeline is provided in supplementary figure 1. 1.

*MRI acquisition*

1. Prior to MRI, each spinal cord specimen is sectioned midline for the whole sagittal length in two halves with a surgical blade (supplementary figure 1. 2).
2. Four tissue chunks are positioned in the four slots of a syringe plunger (supplementary figure 1. 3) and tied with VELCRO®. Medical bandage is also used for padding.
3. An experiment reference frame is defined (sketch in supplementary figure 4), identifying *anterior*, *posterior*, *top* and *bottom* parts of the plunger.
4. Holes made with fine point scissors in the plunger are exploited for radiographic reference (one hole made to the top, anterior part of the plunger; two holes made to the bottom, left part of the plunger).
5. The plunger is fitted into a FALCON® tube, which is finally filled with freshly prepared 10 mM phosphate buffered saline solution for MRI acquisition.
6. Samples were scanned on a 9.4 T Agilent system (Agilent Technologies, Santa Clara, CA, USA; maximum gradient strength/slew rate of $1\,\mathrm{T\,m^{-1}}$ / $6.7\,\mathrm{KT\,m^{-1}\,s^{-1}}$) in two sessions, using a 33 mm diameter coil (Rapid Biomedical GmbH, Rimpar, Germany) for imaging. Each MRI session took an overnight, during which temperature was monitored and kept stable to 35° C using a temperature probe and an MRI compatible air heater (Small Animals Instruments, Inc., Stony Brook, NY, USA).

*Histological procedures*

1. After MRI, standard histological pre-processing is performed (dehydration and subsequent paraffin-embedding).

2. The tissue shrinkage caused by the paraffin-embedding process is estimated. Practically, we measure the size of the specimen along the left-right direction before and after the embedding procedure.

3. Histological sections are sliced sagittally from the surface exposed by the midsagittal cut, consistently with the experiment reference frame introduced prior to MRI.

4. The MRI slice from which each sagittal section is taken is inferred recording the amount of material removed from the exposed surface while looking at an axial MRI view (example in supplementary figure 1. 5), onto which the sagittal slices have been overlaid. Secifically, the thickness of the sagittal MRI slices is scaled to account for tissue shrinkage.

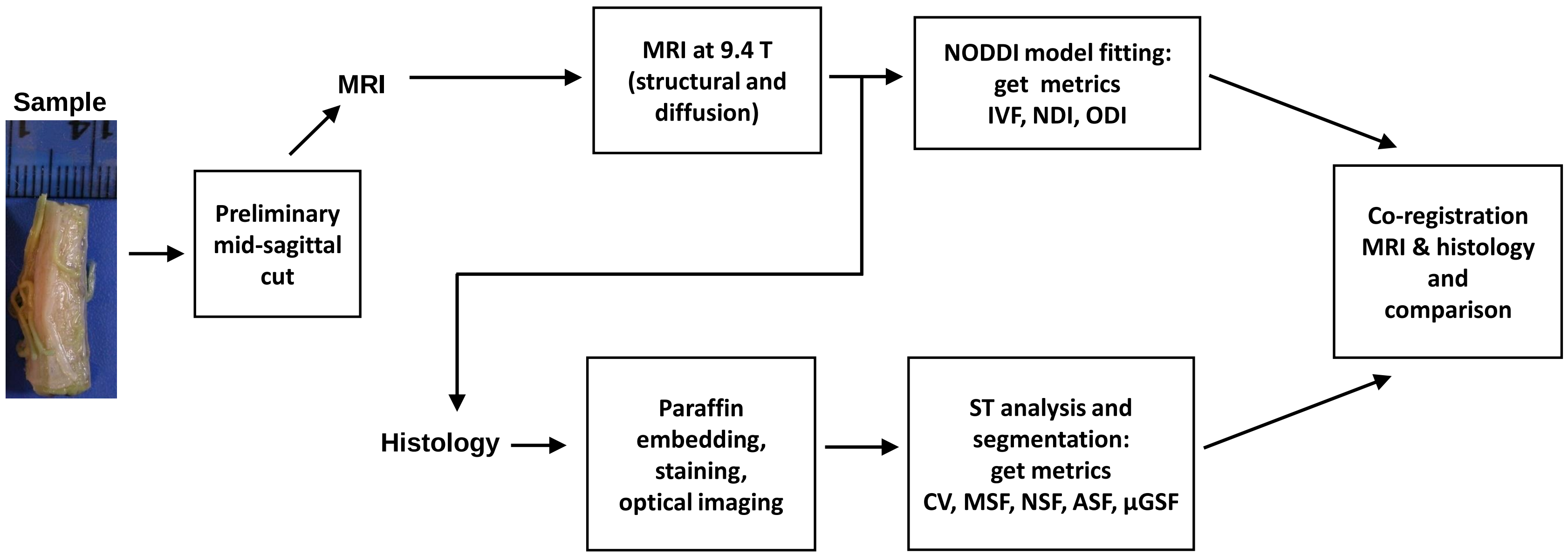


Supplementary figure 1. 1: block diagram summarising our MRI-histology pipeline.

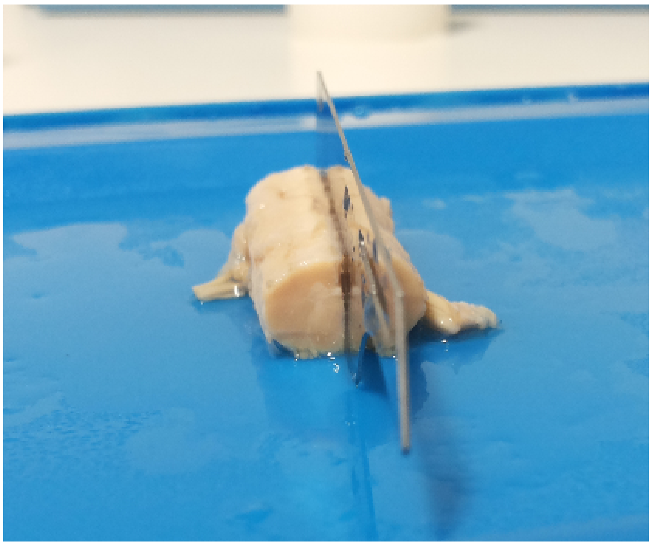

Supplementary figure 1. 2: example of preliminary mid-sagittal cut performed with a surgical blade on the upper lumbar control case.

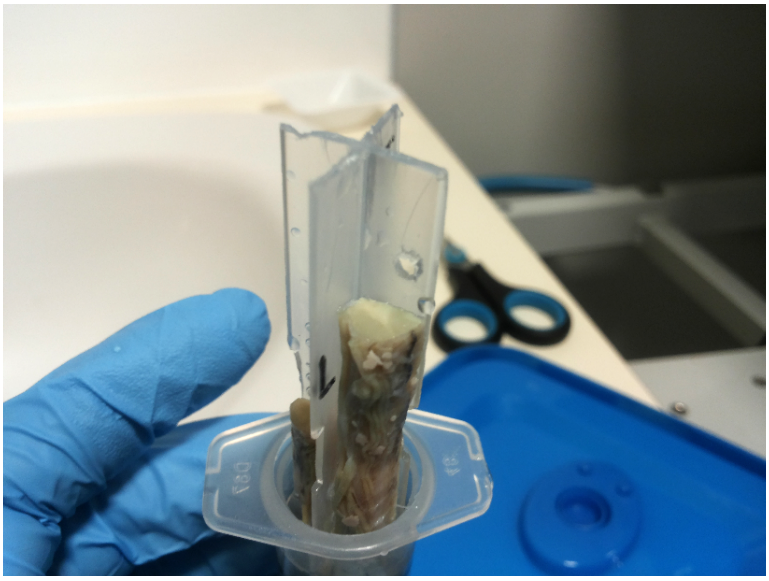

Supplementary figure 1. 3: four chunks of spinal cord tissue being positioned within the four slots of a syringe plunger for MR imaging. Letters A, P, L, R, T and B written with a non-erasable highlighter on the plunger describe the experiment reference frame.

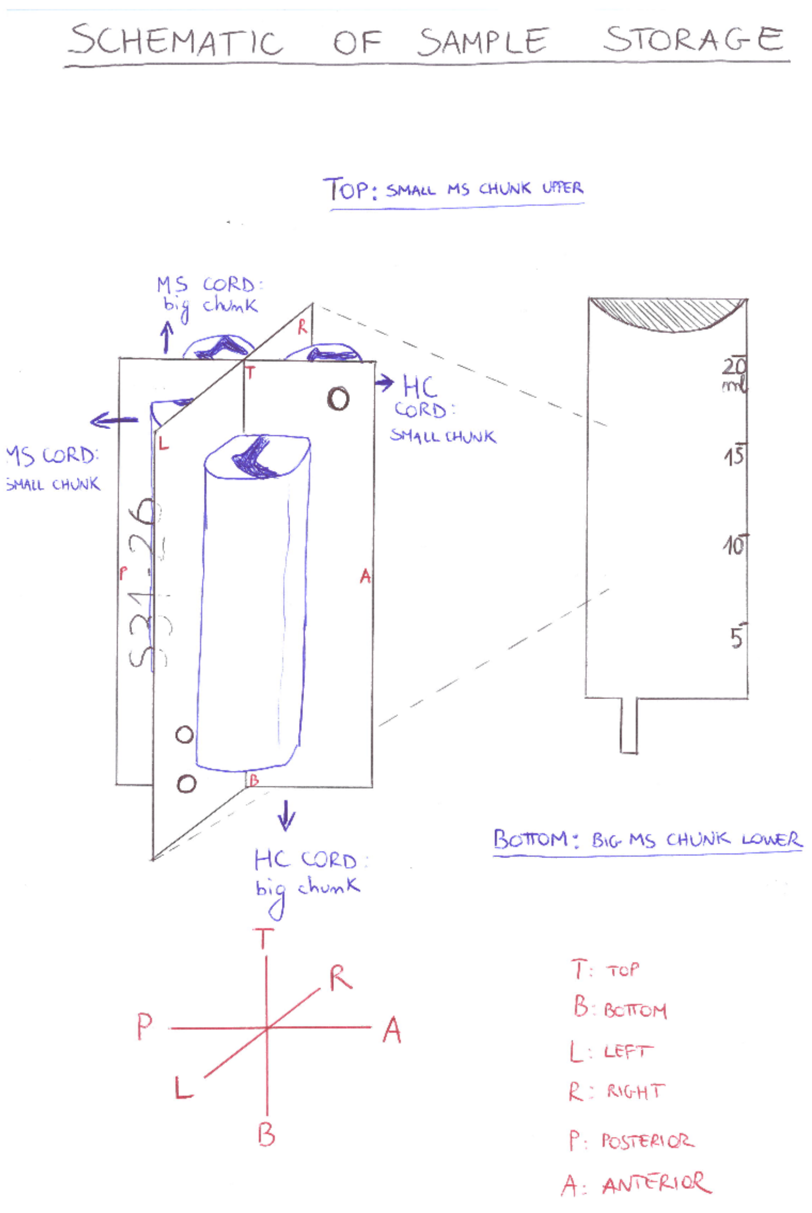


Supplementary figure 1. 4: manual sketch of the experiment reference frame relative to the first of the two MRI sessions.

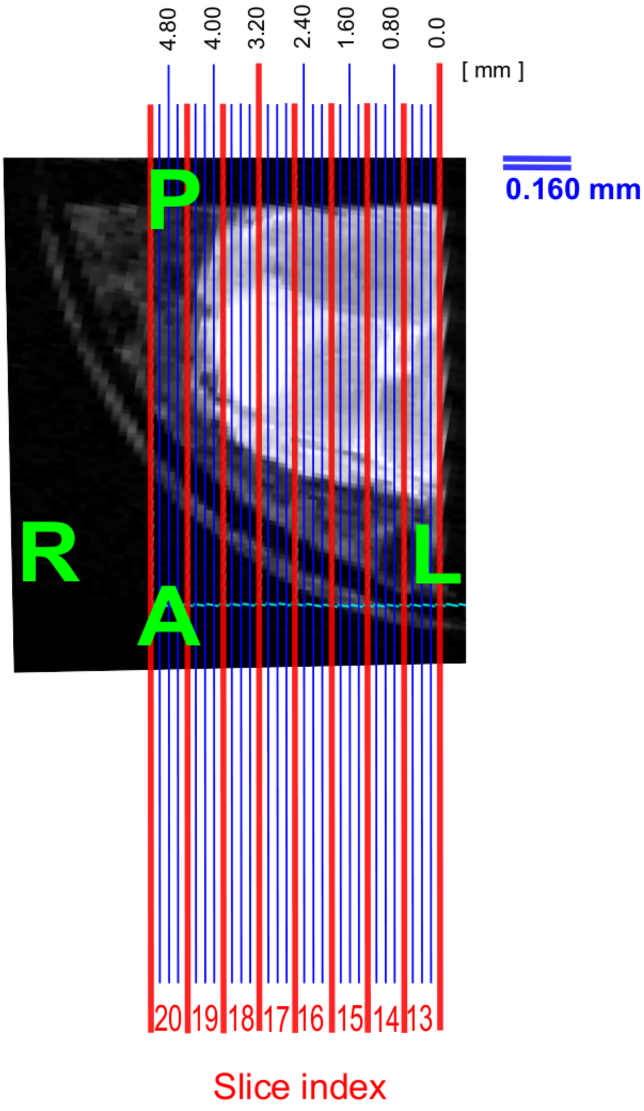


Supplementary figure 1. 5: example of axial MRI views from which the position of the histological sections in the sagittal diffusion-weighted MRI slices was inferred. The thickness of the sagittal slices has been scaled to account for tissue shrinkage due to the paraffin-embedding process. The image shows the upper thoracic MS case (grey matter, non-focal white matter and a white matter focal lesion are visible). Letters A, P, R and L describe the experiment reference frame.
