# Supplementary material for: Neurite dispersion: a new marker of multiple sclerosis spinal cord pathology?
Source: Ann Clin Transl Neurol. 2017 Aug 15;4(9):663–79. doi: 10.1002/acn3.445 (PMC5590517; doi:10.1002/acn3.445)
Supplement: Supplementary file 2 — Data S2. Examples of histological images. [file ACN3-4-663-s002.pdf]

Supplementary material 2: examples of histological images

In this supplementary material, we report examples of the histological images that were obtained from the four tissue specimens.

Control case, upper thoracic level

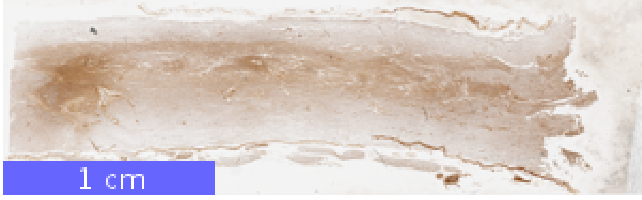

Supplementary figure 2. 1: Palmgren's silver staining, control case, upper thoracic level.

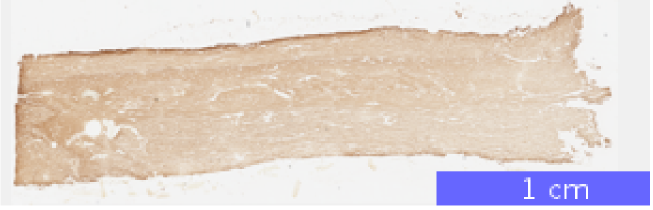

Supplementary figure 2. 2: PLP immunostain, control case, upper thoracic level.

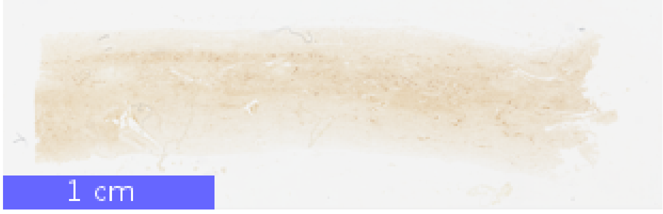

Supplementary figure 2. 3: neurofilament immunostain, control case, upper thoracic level.

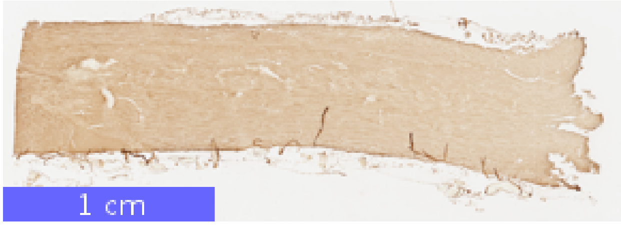

Supplementary figure 2. 4: GFAP immunostain, control case, upper thoracic level.

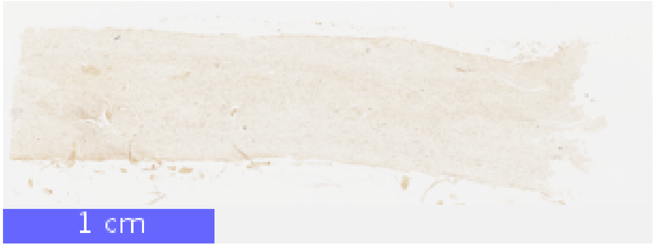

Supplementary figure 2. 5: Iba1 immunostain, control case, upper thoracic level.

Control case, upper lumbar level

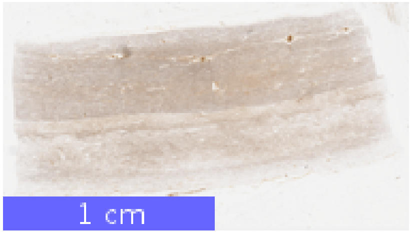

Supplementary figure 2. 6: Palmgren's silver staining, control case, upper lumbar level.

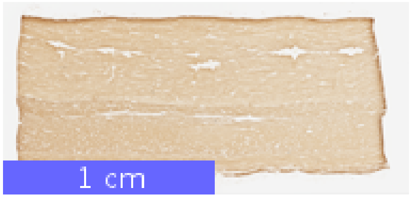

Supplementary figure 2. 7: PLP immunostain, control case, upper lumbar level.

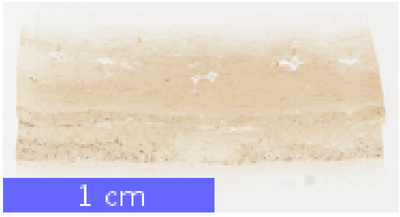

Supplementary figure 2. 8: neurofilament immunostain, control case, upper lumbar level.

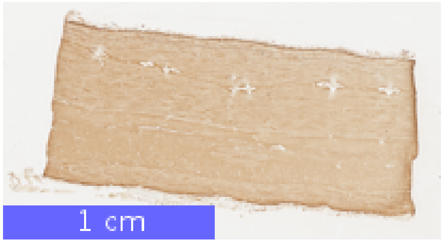

Supplementary figure 2. 9: GFAP immunostain, control case, upper lumbar level.

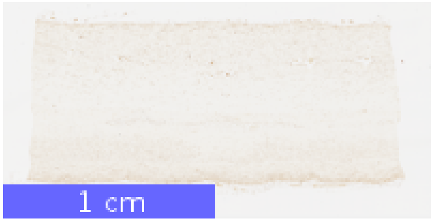

Supplementary figure 2. 10: Iba1 immunostain, control case, upper lumbar level.

Multiple sclerosis case, upper thoracic level

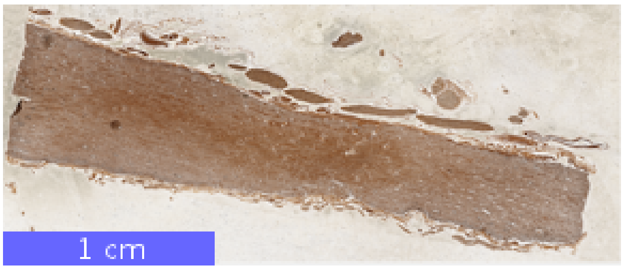

Supplementary figure 2. 11: Palmgren's silver staining, multiple sclerosis case, upper thoracic level.

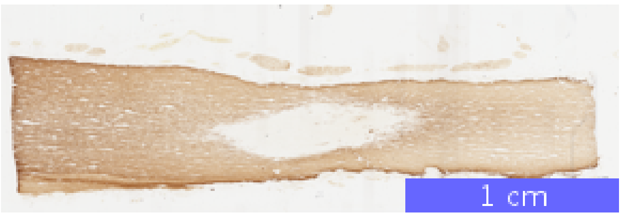

Supplementary figure 2. 12: PLP immunostain, multiple sclerosis case, upper thoracic level.

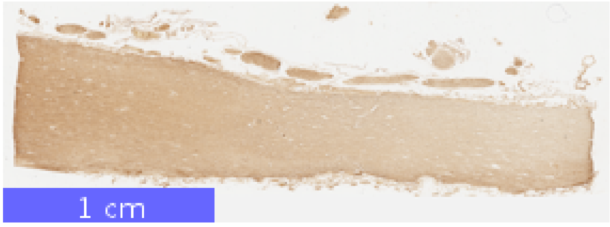

Supplementary figure 2. 13: neurofilament immunostain, multiple sclerosis case, upper thoracic level.

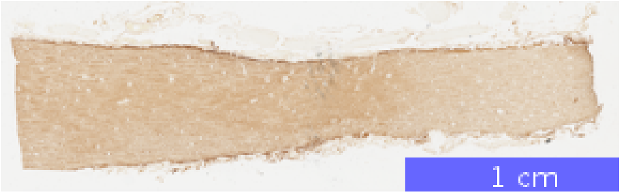

Supplementary figure 2. 14: GFAP immunostain, multiple sclerosis case, upper thoracic level.

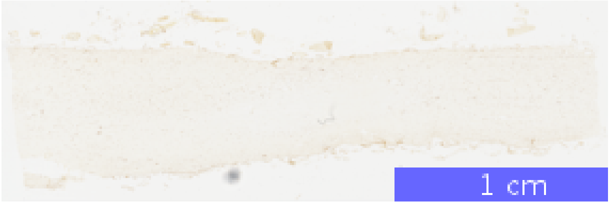

Supplementary figure 2. 15: Iba1 immunostain, multiple sclerosis case, upper thoracic level.

Multiple sclerosis case, upper lumbar level

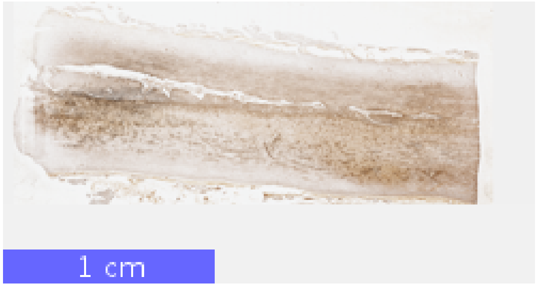

Supplementary figure 2. 16: Palmgren's silver staining, multiple sclerosis case, upper lumbar level.

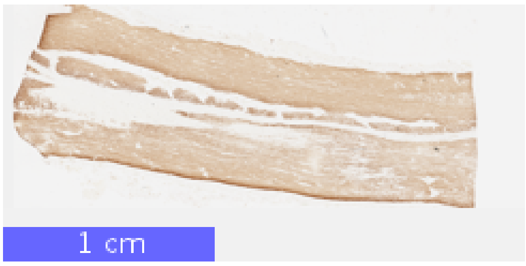

Supplementary figure 2. 17: PLP immunostain, multiple sclerosis case, upper lumbar level.

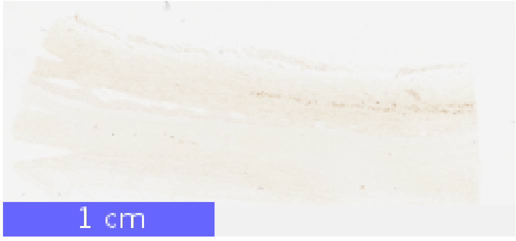

Supplementary figure 2. 18: neurofilament immunostain, multiple sclerosis case, upper lumbar level.

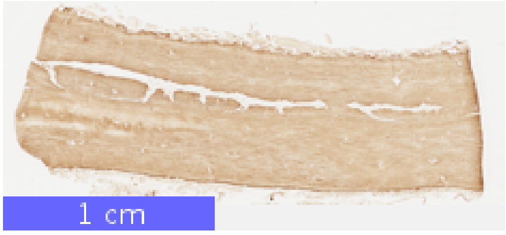

Supplementary figure 2. 19: GFAP immunostain, multiple sclerosis case, upper lumbar level.

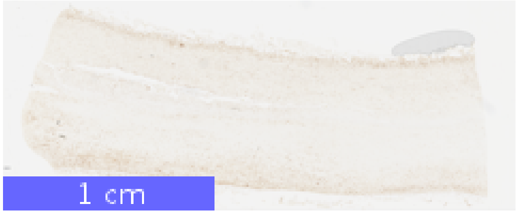

Supplementary figure 2. 20: Iba1 immunostain, multiple sclerosis case, upper lumbar level.
