# Supplementary material for: Neurite dispersion: a new marker of multiple sclerosis spinal cord pathology?
Source: Ann Clin Transl Neurol. 2017 Aug 15;4(9):663–79. doi: 10.1002/acn3.445 (PMC5590517; doi:10.1002/acn3.445)
Supplement: Supplementary file 3 — Data S3. Full results from linear regression models. [file ACN3-4-663-s003.pdf]

SUPPLEMENTARY MATERIAL 3:
FULL RESULTS FROM LINEAR REGRESSION MODELS

```
> ##########  MODEL 1: CONTROLS
> #
> #


> summary(ivf_model1_HC)
Call:
lm(formula = ivf ~ wcv + msfrac + nffrac + gfapfrac + ibafrac,
    data = data_controls)

Residuals:
     Min       1Q   Median       3Q      Max
-0.05412 -0.02567 -0.01802  0.01669  0.09290

Coefficients:
            Estimate Std. Error t value Pr(>|t|)
(Intercept)  0.15026    0.14570   1.031   0.3199
wcv          0.07749    0.05987   1.294   0.2165
msfrac       0.39498    0.19039   2.075   0.0569 .
nffrac       0.13096    0.11127   1.177   0.2588
gfapfrac    -0.89722    0.43194  -2.077   0.0567 .
ibafrac      0.67135    0.67872   0.989   0.3394
---
Signif. codes:  0 ‘***’ 0.001 ‘**’ 0.01 ‘*’ 0.05 ‘.’ 0.1 ‘ ’ 1

Residual standard error: 0.04993 on 14 degrees of freedom
Multiple R-squared:  0.5078,    Adjusted R-squared:  0.332
F-statistic: 2.889 on 5 and 14 DF,  p-value: 0.05371
> # Standardised beta coefficients
> standardCoefs(ivf_model1_HC)
                  b       beta
wcv       0.07749014  0.2510662
msfrac    0.39497600  0.4167176
nffrac    0.13096428  0.2325335
gfapfrac -0.89721912 -0.4075425
ibafrac   0.67135419  0.2020404



> #
> #
> summary(ndi_model1_HC)
Call:
lm(formula = ndi ~ wcv + msfrac + nffrac + gfapfrac + ibafrac,
    data = data_controls)

Residuals:
     Min       1Q   Median       3Q      Max
-0.19269 -0.12787  0.01159  0.09737  0.18818

Coefficients:
            Estimate Std. Error t value Pr(>|t|)
(Intercept)  0.62576    0.43642   1.434 0.173571
wcv         -0.83221    0.17932  -4.641 0.000382 ***
msfrac       0.45880    0.57027   0.805 0.434541
```

```
nffrac      -0.05188    0.33330  -0.156 0.878521
gfapfrac     1.08752    1.29380   0.841 0.414718
ibafrac     -1.42250    2.03302  -0.700 0.495579
---
Signif. codes:  0 ‘***’ 0.001 ‘**’ 0.01 ‘*’ 0.05 ‘.’ 0.1 ‘ ’ 1

Residual standard error: 0.1495 on 14 degrees of freedom
Multiple R-squared:  0.6253,	Adjusted R-squared:  0.4915 
F-statistic: 4.673 on 5 and 14 DF,  p-value: 0.01019
> # Standardised beta coefficients
> standardCoefs(ndi_model1_HC)
                   b        beta
wcv      -0.83221184 -0.78541385
msfrac    0.45880335  0.14100047
nffrac   -0.05188174 -0.02683305
gfapfrac  1.08752470  0.14389190
ibafrac  -1.42249562 -0.12469822



> #
> #
> summary(odi_model1_HC)
Call:
lm(formula = odi ~ wcv + msfrac + nffrac + gfapfrac + ibafrac, 
    data = data_controls)

Residuals:
      Min        1Q    Median        3Q       Max 
-0.073254 -0.023033 -0.008333  0.017822  0.125030 

Coefficients:
            Estimate Std. Error t value Pr(>|t|)    
(Intercept)  0.24676    0.14487   1.703    0.111    
wcv          0.42300    0.05952   7.106 5.28e-06 ***
msfrac      -0.00650    0.18930  -0.034    0.973    
nffrac      -0.14232    0.11064  -1.286    0.219    
gfapfrac     0.21319    0.42947   0.496    0.627    
ibafrac     -1.14437    0.67485  -1.696    0.112    
---
Signif. codes:  0 ‘***’ 0.001 ‘**’ 0.01 ‘*’ 0.05 ‘.’ 0.1 ‘ ’ 1

Residual standard error: 0.04964 on 14 degrees of freedom
Multiple R-squared:  0.8082,	Adjusted R-squared:  0.7397 
F-statistic:  11.8 on 5 and 14 DF,  p-value: 0.000129
> # Standardised beta coefficients
> standardCoefs(odi_model1_HC)
                    b         beta
wcv       0.422997230  0.860404969
msfrac   -0.006500324 -0.004305561
nffrac   -0.142318836 -0.158642139
gfapfrac  0.213195204  0.060796051
ibafrac  -1.144370236 -0.216210415
```

```
> #
> #
> summary(fa_model1_HC)
Call:
lm(formula = fa ~ wcv + msfrac + nffrac + gfapfrac + ibafrac,
    data = data_controls)

Residuals:
     Min       1Q   Median       3Q      Max
-0.22040 -0.03995  0.03243  0.04841  0.07790

Coefficients:
            Estimate Std. Error t value Pr(>|t|)
(Intercept)  0.42545    0.25394   1.675 0.116041
wcv         -0.51019    0.10434  -4.890 0.000239 ***
msfrac      -0.47541    0.33183  -1.433 0.173899
nffrac      -0.01578    0.19394  -0.081 0.936297
gfapfrac     0.64552    0.75284   0.857 0.405638
ibafrac      2.41020    1.18298   2.037 0.060973 .
---
Signif. codes:  0 ‘***’ 0.001 ‘**’ 0.01 ‘*’ 0.05 ‘.’ 0.1 ‘ ’ 1

Residual standard error: 0.08702 on 14 degrees of freedom
Multiple R-squared:  0.6817,    Adjusted R-squared:  0.568
F-statistic: 5.996 on 5 and 14 DF,  p-value: 0.003616
> # Standardised beta coefficients
> standardCoefs(fa_model1_HC)
                  b        beta
wcv      -0.51019002 -0.76269617
msfrac   -0.47541173 -0.23142955
nffrac   -0.01578151 -0.01292882
gfapfrac  0.64552208  0.13528924
ibafrac   2.41019624  0.33466992



> #
> #
> summary(ad_model1_HC)
Call:
lm(formula = ad ~ wcv + msfrac + nffrac + gfapfrac + ibafrac,
    data = data_controls)

Residuals:
     Min       1Q   Median       3Q      Max
-0.17680 -0.11628 -0.02447  0.06506  0.25146

Coefficients:
            Estimate Std. Error t value Pr(>|t|)
(Intercept)   0.6378     0.4442   1.436   0.1730
wcv           0.4856     0.1825   2.661   0.0186 *
msfrac        0.1360     0.5804   0.234   0.8181
nffrac        0.2121     0.3392   0.625   0.5419
gfapfrac     -1.7775     1.3168  -1.350   0.1985
ibafrac       6.0201     2.0692   2.909   0.0114 *
---
Signif. codes:  0 ‘***’ 0.001 ‘**’ 0.01 ‘*’ 0.05 ‘.’ 0.1 ‘ ’ 1

Residual standard error: 0.1522 on 14 degrees of freedom
```

```
Multiple R-squared:  0.6106,	Adjusted R-squared:  0.4715 
F-statistic:  4.39 on 5 and 14 DF,  p-value: 0.01296
> # Standardised beta coefficients
> standardCoefs(ad_model1_HC)
                  b        beta
wcv       0.4856268  0.45906114
msfrac    0.1360051  0.04186513
nffrac    0.2121100  0.10988028
gfapfrac -1.7775107 -0.23556603
ibafrac   6.0200526  0.52858257



> #
> #
> summary(rd_model1_HC)
Call:
lm(formula = rd ~ wcv + msfrac + nffrac + gfapfrac + ibafrac, 
    data = data_controls)

Residuals:
     Min       1Q   Median       3Q      Max 
-0.13930 -0.07470 -0.02109  0.05208  0.22582 

Coefficients:
            Estimate Std. Error t value Pr(>|t|)   
(Intercept)   0.3786     0.3707   1.021  0.32449   
wcv           0.5587     0.1523   3.667  0.00254 **
msfrac        0.4244     0.4845   0.876  0.39580   
nffrac        0.2357     0.2831   0.832  0.41914   
gfapfrac     -1.6289     1.0991  -1.482  0.16048   
ibafrac       2.1048     1.7271   1.219  0.24310   
---
Signif. codes:  0 ‘***’ 0.001 ‘**’ 0.01 ‘*’ 0.05 ‘.’ 0.1 ‘ ’ 1

Residual standard error: 0.127 on 14 degrees of freedom
Multiple R-squared:  0.6036,	Adjusted R-squared:  0.4621 
F-statistic: 4.264 on 5 and 14 DF,  p-value: 0.01447
> # Standardised beta coefficients
> standardCoefs(rd_model1_HC)
                  b       beta
wcv       0.5586765  0.6383478
msfrac    0.4243925  0.1579043
nffrac    0.2356963  0.1475844
gfapfrac -1.6289263 -0.2609342
ibafrac   2.1047709  0.2233811



> #
> #
> summary(md_model1_HC)
Call:
lm(formula = md ~ wcv + msfrac + nffrac + gfapfrac + ibafrac, 
    data = data_controls)

Residuals:
     Min       1Q   Median       3Q      Max 
-0.14553 -0.07667 -0.02743  0.05765  0.19739 
```

```
Coefficients:
            Estimate Std. Error t value Pr(>|t|)
(Intercept)   0.4533     0.3608   1.256   0.2295
wcv           0.5135     0.1482   3.464   0.0038 **
msfrac        0.2848     0.4714   0.604   0.5555
nffrac        0.2099     0.2755   0.762   0.4589
gfapfrac     -1.5801     1.0696  -1.477   0.1617
ibafrac       3.3560     1.6807   1.997   0.0657 .
---
Signif. codes:  0 ‘***’ 0.001 ‘**’ 0.01 ‘*’ 0.05 ‘.’ 0.1 ‘ ’ 1

Residual standard error: 0.1236 on 14 degrees of freedom
Multiple R-squared:   0.62,     Adjusted R-squared:  0.4842
F-statistic: 4.568 on 5 and 14 DF,  p-value: 0.01113
> # Standardised beta coefficients
> standardCoefs(md_model1_HC)
                  b       beta
wcv       0.5134829  0.5903590
msfrac    0.2847750  0.1066158
nffrac    0.2098885  0.1322421
gfapfrac -1.5801123 -0.2546893
ibafrac   3.3560108  0.3583920


> ##########  MODEL 1: MS
> #
> #

> summary(ivf_model1_MS)
Call:
lm(formula = ivf ~ wcv + msfrac + nffrac + gfapfrac + ibafrac,
    data = data_multiple_sclerosis)

Residuals:
     Min       1Q   Median       3Q      Max
-0.10485 -0.04748 -0.01853  0.04332  0.18340

Coefficients:
            Estimate Std. Error t value Pr(>|t|)
(Intercept)  0.24755    0.09869   2.508   0.0200 *
wcv         -0.08227    0.09362  -0.879   0.3890
msfrac      -0.29601    0.16054  -1.844   0.0787 .
nffrac       0.10977    0.17280   0.635   0.5318
gfapfrac    -0.35039    0.19013  -1.843   0.0789 .
ibafrac     -0.31606    0.45558  -0.694   0.4951
---
Signif. codes:  0 ‘***’ 0.001 ‘**’ 0.01 ‘*’ 0.05 ‘.’ 0.1 ‘ ’ 1

Residual standard error: 0.07482 on 22 degrees of freedom
Multiple R-squared:  0.2045,    Adjusted R-squared:  0.02366
F-statistic: 1.131 on 5 and 22 DF,  p-value: 0.3735
> # Standardised beta coefficients
> standardCoefs(ivf_model1_MS)
                   b       beta
wcv      -0.08227353 -0.1926276
msfrac   -0.29601342 -0.4373360
nffrac    0.10976930  0.1876239
gfapfrac -0.35038577 -0.4779905
```

```
ibafrac  -0.31606390 -0.1929732



> #
> #
> summary(ndi_model1_MS)
Call:
lm(formula = ndi ~ wcv + msfrac + nffrac + gfapfrac + ibafrac,
    data = data_multiple_sclerosis)

Residuals:
     Min       1Q   Median       3Q      Max
-0.42076 -0.11052 -0.04117  0.12519  0.31868

Coefficients:
            Estimate Std. Error t value Pr(>|t|)
(Intercept)   0.1679     0.2700   0.622 0.540280
wcv          -0.4074     0.2561  -1.591 0.125934
msfrac        1.9988     0.4392   4.552 0.000157 ***
nffrac        0.2316     0.4727   0.490 0.628947
gfapfrac      0.4142     0.5201   0.796 0.434289
ibafrac       1.1817     1.2462   0.948 0.353283
---
Signif. codes:  0 ‘***’ 0.001 ‘**’ 0.01 ‘*’ 0.05 ‘.’ 0.1 ‘ ’ 1

Residual standard error: 0.2047 on 22 degrees of freedom
Multiple R-squared:  0.687,     Adjusted R-squared:  0.6159
F-statistic: 9.658 on 5 and 22 DF,  p-value: 5.483e-05
> # Standardised beta coefficients
> standardCoefs(ndi_model1_MS)
                 b       beta
wcv      -0.4073905 -0.2187166
msfrac    1.9988116  0.6771551
nffrac    0.2316374  0.0907880
gfapfrac  0.4142172  0.1295727
ibafrac   1.1817497  0.1654475



> #
> #
> summary(odi_model1_MS)
Call:
lm(formula = odi ~ wcv + msfrac + nffrac + gfapfrac + ibafrac,
    data = data_multiple_sclerosis)

Residuals:
      Min        1Q    Median        3Q       Max
-0.130467 -0.052017  0.002505  0.051527  0.104710

Coefficients:
            Estimate Std. Error t value Pr(>|t|)
(Intercept)  0.01956    0.08849   0.221    0.827
wcv          0.41708    0.08394   4.969 5.68e-05 ***
msfrac       0.23469    0.14394   1.630    0.117
nffrac       0.41885    0.15493   2.703    0.013 *
gfapfrac     0.04865    0.17047   0.285    0.778
ibafrac     -0.49001    0.40847  -1.200    0.243
```

```
---
Signif. codes:  0 ‘***’ 0.001 ‘**’ 0.01 ‘*’ 0.05 ‘.’ 0.1 ‘ ’ 1

Residual standard error: 0.06709 on 22 degrees of freedom
Multiple R-squared:  0.6613,    Adjusted R-squared:  0.5843
F-statistic:  8.59 on 5 and 22 DF,  p-value: 0.0001243
> # Standardised beta coefficients
> standardCoefs(odi_model1_MS)
                   b        beta
wcv       0.41708328  0.71067230
msfrac    0.23469167  0.25234215
nffrac    0.41884872  0.52101814
gfapfrac  0.04864748  0.04829714
ibafrac  -0.49001212 -0.21772949



> #
> #
> summary(fa_model1_MS)
Call:
lm(formula = fa ~ wcv + msfrac + nffrac + gfapfrac + ibafrac,
    data = data_multiple_sclerosis)

Residuals:
     Min       1Q   Median       3Q      Max
-0.28667 -0.17121 -0.02764  0.15294  0.34193

Coefficients:
            Estimate Std. Error t value Pr(>|t|)
(Intercept)  0.25789    0.25625   1.006    0.325
wcv         -0.08198    0.24309  -0.337    0.739
msfrac       0.51887    0.41685   1.245    0.226
nffrac       0.06332    0.44867   0.141    0.889
gfapfrac    -0.10529    0.49368  -0.213    0.833
ibafrac      1.05439    1.18290   0.891    0.382

Residual standard error: 0.1943 on 22 degrees of freedom
Multiple R-squared:  0.2452,    Adjusted R-squared:  0.07366
F-statistic: 1.429 on 5 and 22 DF,  p-value: 0.2529
> # Standardised beta coefficients
> standardCoefs(fa_model1_MS)
                   b        beta
wcv      -0.08197811 -0.07200420
msfrac    0.51886502  0.28758075
nffrac    0.06332446  0.04060509
gfapfrac -0.10528691 -0.05388263
ibafrac   1.05439215  0.24150487



> #
> #
> summary(ad_model1_MS)
Call:
lm(formula = ad ~ wcv + msfrac + nffrac + gfapfrac + ibafrac,
    data = data_multiple_sclerosis)

Residuals:
```

```
     Min       1Q   Median       3Q      Max 
-0.45061 -0.18123 -0.00208  0.11087  0.54071 

Coefficients:
            Estimate Std. Error t value Pr(>|t|)    
(Intercept)   1.7779     0.3653   4.867 7.27e-05 ***
wcv          -0.1706     0.3465  -0.492 0.627425    
msfrac       -2.5924     0.5942  -4.363 0.000249 ***
nffrac       -0.7575     0.6396  -1.184 0.248891    
gfapfrac     -0.8645     0.7038  -1.228 0.232289    
ibafrac      -2.0818     1.6863  -1.235 0.230011    
---
Signif. codes:  0 ‘***’ 0.001 ‘**’ 0.01 ‘*’ 0.05 ‘.’ 0.1 ‘ ’ 1

Residual standard error: 0.2769 on 22 degrees of freedom
Multiple R-squared:  0.7066,    Adjusted R-squared:  0.6399 
F-statistic:  10.6 on 5 and 22 DF,  p-value: 2.798e-05
> # Standardised beta coefficients
> standardCoefs(ad_model1_MS)
                  b        beta
wcv      -0.1705805 -0.06553108
msfrac   -2.5924364 -0.62845106
nffrac   -0.7575437 -0.21245874
gfapfrac -0.8644660 -0.19349970
ibafrac  -2.0818402 -0.20855906




> #
> #
> summary(rd_model1_MS)
Call:
lm(formula = rd ~ wcv + msfrac + nffrac + gfapfrac + ibafrac, 
    data = data_multiple_sclerosis)

Residuals:
     Min       1Q   Median       3Q      Max 
-0.45004 -0.23725  0.02189  0.16744  0.74146 

Coefficients:
            Estimate Std. Error t value Pr(>|t|)   
(Intercept)   1.4012     0.4460   3.141  0.00474 **
wcv          -0.1737     0.4231  -0.411  0.68538   
msfrac       -1.9533     0.7256  -2.692  0.01332 * 
nffrac       -0.7120     0.7810  -0.912  0.37184   
gfapfrac     -0.8204     0.8593  -0.955  0.35008   
ibafrac      -1.9581     2.0591  -0.951  0.35195   
---
Signif. codes:  0 ‘***’ 0.001 ‘**’ 0.01 ‘*’ 0.05 ‘.’ 0.1 ‘ ’ 1

Residual standard error: 0.3382 on 22 degrees of freedom
Multiple R-squared:  0.5194,    Adjusted R-squared:  0.4102 
F-statistic: 4.756 on 5 and 22 DF,  p-value: 0.004265
> # Standardised beta coefficients
> standardCoefs(rd_model1_MS)
                  b        beta
wcv      -0.1737172 -0.06994211
msfrac   -1.9532896 -0.49625851
nffrac   -0.7119917 -0.20927616
```

```
gfapfrac -0.8204378 -0.19246687
ibafrac  -1.9581213 -0.20558870



> #
> #
> summary(md_model1_MS)
Call:
lm(formula = md ~ wcv + msfrac + nffrac + gfapfrac + ibafrac,
    data = data_multiple_sclerosis)

Residuals:
     Min       1Q   Median       3Q      Max
-0.41830 -0.22987 -0.01078  0.12651  0.66763

Coefficients:
            Estimate Std. Error t value Pr(>|t|)
(Intercept)   1.5188     0.4066   3.736  0.00115 **
wcv          -0.1634     0.3857  -0.424  0.67584
msfrac       -2.1414     0.6614  -3.238  0.00378 **
nffrac       -0.7662     0.7119  -1.076  0.29342
gfapfrac     -0.8221     0.7833  -1.050  0.30530
ibafrac      -1.9713     1.8768  -1.050  0.30494
---
Signif. codes:  0 ‘***’ 0.001 ‘**’ 0.01 ‘*’ 0.05 ‘.’ 0.1 ‘ ’ 1

Residual standard error: 0.3082 on 22 degrees of freedom
Multiple R-squared:  0.601,	Adjusted R-squared:  0.5103
F-statistic: 6.628 on 5 and 22 DF,  p-value: 0.0006646
> # Standardised beta coefficients
> standardCoefs(md_model1_MS)
                  b        beta
wcv      -0.1634456 -0.06578528
msfrac   -2.1414243 -0.54388086
nffrac   -0.7662329 -0.22514658
gfapfrac -0.8221089 -0.19279661
ibafrac  -1.9713285 -0.20690852
```

```
> ##########  MODEL 2: CONTROLS


> #
> #
> summary(ivf_model2_HC)
Call:
lm(formula = ivf ~ wcv + msfrac + nffrac, data = data_controls)

Residuals:
      Min        1Q    Median        3Q       Max 
-0.056495 -0.031848 -0.014634  0.005901  0.101303 

Coefficients:
            Estimate Std. Error t value Pr(>|t|)  
(Intercept) -0.13275    0.05747  -2.310   0.0346 *
wcv          0.07488    0.06430   1.164   0.2613  
msfrac       0.34605    0.19746   1.752   0.0988 .
nffrac       0.18180    0.11550   1.574   0.1350  
---
Signif. codes:  0 ‘***’ 0.001 ‘**’ 0.01 ‘*’ 0.05 ‘.’ 0.1 ‘ ’ 1

Residual standard error: 0.05427 on 16 degrees of freedom
Multiple R-squared:  0.3354,    Adjusted R-squared:  0.2108 
F-statistic: 2.691 on 3 and 16 DF,  p-value: 0.08106
> # Standardised beta coefficients
> standardCoefs(ivf_model2_HC)
               b      beta
wcv    0.0748756 0.2425952
msfrac 0.3460469 0.3650952
nffrac 0.1818004 0.3227954



> #
> #
> summary(ndi_model2_HC)
Call:
lm(formula = ndi ~ wcv + msfrac + nffrac, data = data_controls)

Residuals:
     Min       1Q   Median       3Q      Max 
-0.23728 -0.10119  0.01995  0.10064  0.20230 

Coefficients:
            Estimate Std. Error t value Pr(>|t|)    
(Intercept)   0.9700     0.1538   6.308 1.04e-05 ***
wcv          -0.8364     0.1720  -4.862 0.000173 ***
msfrac        0.4806     0.5283   0.910 0.376480    
nffrac       -0.1408     0.3090  -0.456 0.654814    
---
Signif. codes:  0 ‘***’ 0.001 ‘**’ 0.01 ‘*’ 0.05 ‘.’ 0.1 ‘ ’ 1

Residual standard error: 0.1452 on 16 degrees of freedom
Multiple R-squared:  0.5964,    Adjusted R-squared:  0.5207 
F-statistic:  7.88 on 3 and 16 DF,  p-value: 0.001882
> # Standardised beta coefficients
> standardCoefs(ndi_model2_HC)
                b        beta
```

```
wcv    -0.8363835 -0.78935088
msfrac  0.4805778  0.14769224
nffrac -0.1407752 -0.07280844



> #
> #
> summary(odi_model2_HC)
Call:
lm(formula = odi ~ wcv + msfrac + nffrac, data = data_controls)

Residuals:
      Min        1Q    Median        3Q       Max 
-0.072079 -0.033167  0.004971  0.025464  0.124652 

Coefficients:
            Estimate Std. Error t value Pr(>|t|)    
(Intercept)  0.31593    0.05415   5.835 2.54e-05 ***
wcv          0.41174    0.06058   6.796 4.29e-06 ***
msfrac      -0.05560    0.18604  -0.299   0.7689    
nffrac      -0.19852    0.10882  -1.824   0.0868 .  
---
Signif. codes:  0 ‘***’ 0.001 ‘**’ 0.01 ‘*’ 0.05 ‘.’ 0.1 ‘ ’ 1

Residual standard error: 0.05113 on 16 degrees of freedom
Multiple R-squared:  0.7675,    Adjusted R-squared:  0.7239 
F-statistic:  17.6 on 3 and 16 DF,  p-value: 2.541e-05
> # Standardised beta coefficients
> standardCoefs(odi_model2_HC)
                 b        beta
wcv     0.41174246  0.83751200
msfrac -0.05559575 -0.03682445
nffrac -0.19852442 -0.22129424



> #
> #
> summary(fa_model2_HC)
Call:
lm(formula = fa ~ wcv + msfrac + nffrac, data = data_controls)

Residuals:
      Min        1Q    Median        3Q       Max 
-0.223297 -0.052961 -0.001793  0.058757  0.140749 

Coefficients:
            Estimate Std. Error t value Pr(>|t|)    
(Intercept)  0.62343    0.10129   6.155 1.38e-05 ***
wcv         -0.47342    0.11333  -4.177 0.000711 ***
msfrac      -0.26182    0.34801  -0.752 0.462767    
nffrac       0.07727    0.20356   0.380 0.709220    
---
Signif. codes:  0 ‘***’ 0.001 ‘**’ 0.01 ‘*’ 0.05 ‘.’ 0.1 ‘ ’ 1

Residual standard error: 0.09564 on 16 degrees of freedom
Multiple R-squared:  0.5605,    Adjusted R-squared:  0.4781 
F-statistic: 6.802 on 3 and 16 DF,  p-value: 0.003625
```

```
> # Standardised beta coefficients
> standardCoefs(fa_model2_HC)
                 b        beta
wcv    -0.47342026 -0.70772811
msfrac -0.26182380 -0.12745535
nffrac  0.07727417  0.06330598



> #
> #
> summary(ad_model2_HC)
Call:
lm(formula = ad ~ wcv + msfrac + nffrac, data = data_controls)

Residuals:
     Min       1Q   Median       3Q      Max 
-0.23433 -0.12549 -0.04001  0.07075  0.31020 

Coefficients:
            Estimate Std. Error t value Pr(>|t|)  
(Intercept)  0.06798    0.19554   0.348   0.7326  
wcv          0.53700    0.21878   2.455   0.0259 *
msfrac       0.32824    0.67184   0.489   0.6318  
nffrac       0.52296    0.39297   1.331   0.2019  
---
Signif. codes:  0 ‘***’ 0.001 ‘**’ 0.01 ‘*’ 0.05 ‘.’ 0.1 ‘ ’ 1

Residual standard error: 0.1846 on 16 degrees of freedom
Multiple R-squared:  0.3451,	Adjusted R-squared:  0.2223 
F-statistic:  2.81 on 3 and 16 DF,  p-value: 0.07283
> # Standardised beta coefficients
> standardCoefs(ad_model2_HC)
               b      beta
wcv    0.5370029 0.5076268
msfrac 0.3282386 0.1010385
nffrac 0.5229593 0.2709109



> #
> #
> summary(rd_model2_HC)
Call:
lm(formula = rd ~ wcv + msfrac + nffrac, data = data_controls)

Residuals:
     Min       1Q   Median       3Q      Max 
-0.17510 -0.09608 -0.02431  0.04544  0.28809 

Coefficients:
            Estimate Std. Error t value Pr(>|t|)   
(Intercept)  -0.1369     0.1400  -0.978  0.34260   
wcv           0.5646     0.1567   3.604  0.00238 **
msfrac        0.3902     0.4811   0.811  0.42921   
nffrac        0.3677     0.2814   1.307  0.20978   
---
Signif. codes:  0 ‘***’ 0.001 ‘**’ 0.01 ‘*’ 0.05 ‘.’ 0.1 ‘ ’ 1
```

```
Residual standard error: 0.1322 on 16 degrees of freedom
Multiple R-squared:  0.5094,    Adjusted R-squared:  0.4174 
F-statistic: 5.538 on 3 and 16 DF,  p-value: 0.008404
> # Standardised beta coefficients
> standardCoefs(rd_model2_HC)
               b      beta
wcv    0.5646127 0.6451306
msfrac 0.3901822 0.1451756
nffrac 0.3676838 0.2302302



> #
> #
> summary(md_model2_HC)
Call:
lm(formula = md ~ wcv + msfrac + nffrac, data = data_controls)

Residuals:
     Min       1Q   Median       3Q      Max 
-0.18678 -0.07356 -0.02982  0.05331  0.26663 

Coefficients:
            Estimate Std. Error t value Pr(>|t|)   
(Intercept) -0.04934    0.14489  -0.341  0.73790   
wcv          0.53509    0.16211   3.301  0.00451 **
msfrac       0.33263    0.49781   0.668  0.51354   
nffrac       0.39681    0.29118   1.363  0.19183   
---
Signif. codes:  0 ‘***’ 0.001 ‘**’ 0.01 ‘*’ 0.05 ‘.’ 0.1 ‘ ’ 1

Residual standard error: 0.1368 on 16 degrees of freedom
Multiple R-squared:  0.4681,    Adjusted R-squared:  0.3684 
F-statistic: 4.694 on 3 and 16 DF,  p-value: 0.01551
> # Standardised beta coefficients
> standardCoefs(md_model2_HC)
               b      beta
wcv    0.5350902 0.6152013
msfrac 0.3326255 0.1245303
nffrac 0.3968086 0.2500127



> ##########  MODEL 2: MS


> #
> #
> summary(ivf_model2_MS)
Call:
lm(formula = ivf ~ wcv + msfrac + nffrac, data = data_multiple_sclerosis)

Residuals:
     Min       1Q   Median       3Q      Max 
-0.08678 -0.04689 -0.02072  0.01904  0.23148 

Coefficients:
             Estimate Std. Error t value Pr(>|t|)  
(Intercept)  0.093097   0.052706   1.766   0.0901 .
```

```
wcv          -0.005985   0.085885  -0.070   0.9450
msfrac       -0.177201   0.148676  -1.192   0.2450
nffrac       -0.027333   0.130839  -0.209   0.8363
---
Signif. codes:  0 ‘***’ 0.001 ‘**’ 0.01 ‘*’ 0.05 ‘.’ 0.1 ‘ ’ 1

Residual standard error: 0.07697 on 24 degrees of freedom
Multiple R-squared:  0.08165,	Adjusted R-squared:  -0.03314
F-statistic: 0.7113 on 3 and 24 DF,  p-value: 0.5548
> # Standardised beta coefficients
> standardCoefs(ivf_model2_MS)
                  b        beta
wcv    -0.005984537 -0.01401164
msfrac -0.177200658 -0.26179971
nffrac -0.027333048 -0.04671920



> #
> #
> summary(ndi_model2_MS)
Call:
lm(formula = ndi ~ wcv + msfrac + nffrac, data = data_multiple_sclerosis)

Residuals:
     Min       1Q   Median       3Q      Max
-0.44467 -0.10585 -0.03327  0.08482  0.38173

Coefficients:
            Estimate Std. Error t value Pr(>|t|)
(Intercept)   0.3313     0.1376   2.408   0.0241 *
wcv          -0.4798     0.2242  -2.140   0.0427 *
msfrac        1.9109     0.3880   4.925 5.03e-05 ***
nffrac        0.5661     0.3415   1.658   0.1104
---
Signif. codes:  0 ‘***’ 0.001 ‘**’ 0.01 ‘*’ 0.05 ‘.’ 0.1 ‘ ’ 1

Residual standard error: 0.2009 on 24 degrees of freedom
Multiple R-squared:  0.6711,	Adjusted R-squared:   0.63
F-statistic: 16.32 on 3 and 24 DF,  p-value: 5.39e-06
> # Standardised beta coefficients
> standardCoefs(ndi_model2_MS)
                b       beta
wcv    -0.4797959 -0.2575890
msfrac  1.9109297  0.6473826
nffrac  0.5660951  0.2218754



> #
> #
> summary(odi_model2_MS)
Call:
lm(formula = odi ~ wcv + msfrac + nffrac, data = data_multiple_sclerosis)

Residuals:
      Min        1Q    Median        3Q       Max
-0.139583 -0.044075  0.007777  0.043264  0.117817
```

```
Coefficients:
            Estimate Std. Error t value Pr(>|t|)
(Intercept)  0.05371    0.04602   1.167  0.25466
wcv          0.39474    0.07499   5.264 2.13e-05 ***
msfrac       0.18346    0.12982   1.413  0.17044
nffrac       0.32400    0.11425   2.836  0.00913 **
---
Signif. codes:  0 ‘***’ 0.001 ‘**’ 0.01 ‘*’ 0.05 ‘.’ 0.1 ‘ ’ 1

Residual standard error: 0.06721 on 24 degrees of freedom
Multiple R-squared:  0.6292,    Adjusted R-squared:  0.5828
F-statistic: 13.57 on 3 and 24 DF,  p-value: 2.211e-05
> # Standardised beta coefficients
> standardCoefs(odi_model2_MS)
               b      beta
wcv    0.3947435 0.6726074
msfrac 0.1834605 0.1972580
nffrac 0.3239980 0.4030305



> #
> #
> summary(fa_model2_MS)
Call:
lm(formula = fa ~ wcv + msfrac + nffrac, data = data_multiple_sclerosis)

Residuals:
     Min       1Q   Median       3Q      Max
-0.27815 -0.17140  0.01079  0.14338  0.31141

Coefficients:
            Estimate Std. Error t value Pr(>|t|)
(Intercept)  0.18414    0.13067   1.409    0.172
wcv         -0.03376    0.21293  -0.159    0.875
msfrac       0.62934    0.36860   1.707    0.101
nffrac       0.26730    0.32437   0.824    0.418

Residual standard error: 0.1908 on 24 degrees of freedom
Multiple R-squared:  0.2056,    Adjusted R-squared:  0.1063
F-statistic: 2.071 on 3 and 24 DF,  p-value: 0.1308
> # Standardised beta coefficients
> standardCoefs(fa_model2_MS)
                b        beta
wcv    -0.0337635 -0.02965565
msfrac  0.6293449  0.34881415
nffrac  0.2673001  0.17139892



> #
> #
> summary(ad_model2_MS)
Call:
lm(formula = ad ~ wcv + msfrac + nffrac, data = data_multiple_sclerosis)

Residuals:
    Min      1Q  Median      3Q     Max
-0.4627 -0.1494 -0.0224  0.1140  0.5926
```

```
Coefficients:
             Estimate Std. Error t value Pr(>|t|)
(Intercept)  1.42782    0.19041   7.499 9.73e-08 ***
wcv         -0.01101    0.31028  -0.035 0.971982
msfrac      -2.38402    0.53713  -4.438 0.000173 ***
nffrac      -1.37354    0.47268  -2.906 0.007753 **
---
Signif. codes:  0 '***' 0.001 '**' 0.01 '*' 0.05 '.' 0.1 ' ' 1

Residual standard error: 0.2781 on 24 degrees of freedom
Multiple R-squared:  0.6773,    Adjusted R-squared:  0.637
F-statistic: 16.79 on 3 and 24 DF,  p-value: 4.305e-06
> # Standardised beta coefficients
> standardCoefs(ad_model2_MS)
                 b         beta
wcv    -0.01101194 -0.004230403
msfrac -2.38401525 -0.577926212
nffrac -1.37354360 -0.385220462
```

```
> #
> #
> summary(rd_model2_MS)
Call:
lm(formula = rd ~ wcv + msfrac + nffrac, data = data_multiple_sclerosis)

Residuals:
     Min       1Q   Median       3Q      Max
-0.46981 -0.26319  0.02215  0.15040  0.81246

Coefficients:
             Estimate Std. Error t value Pr(>|t|)
(Intercept)  1.06853    0.22824   4.682 9.33e-05 ***
wcv         -0.02189    0.37191  -0.059   0.9536
msfrac      -1.75433    0.64382  -2.725   0.0118 *
nffrac      -1.29284    0.56658  -2.282   0.0317 *
---
Signif. codes:  0 '***' 0.001 '**' 0.01 '*' 0.05 '.' 0.1 ' ' 1

Residual standard error: 0.3333 on 24 degrees of freedom
Multiple R-squared:  0.4907,    Adjusted R-squared:  0.4271
F-statistic: 7.709 on 3 and 24 DF,  p-value: 0.0008919
> # Standardised beta coefficients
> standardCoefs(rd_model2_MS)
                 b         beta
wcv    -0.02188643 -0.008811925
msfrac -1.75433283 -0.445710960
nffrac -1.29284483 -0.380006708
```

```
> #
> #
> summary(md_model2_MS)
Call:
lm(formula = md ~ wcv + msfrac + nffrac, data = data_multiple_sclerosis)

Residuals:
     Min       1Q   Median       3Q      Max 
-0.46421 -0.18669 -0.03164  0.14455  0.73870 

Coefficients:
            Estimate Std. Error t value Pr(>|t|)    
(Intercept)  1.18565    0.20928   5.665  7.8e-06 ***
wcv         -0.01151    0.34103  -0.034  0.97336    
msfrac      -1.94266    0.59036  -3.291  0.00308 ** 
nffrac      -1.35024    0.51953  -2.599  0.01574 *  
---
Signif. codes:  0 ‘***’ 0.001 ‘**’ 0.01 ‘*’ 0.05 ‘.’ 0.1 ‘ ’ 1

Residual standard error: 0.3056 on 24 degrees of freedom
Multiple R-squared:  0.5721,    Adjusted R-squared:  0.5186 
F-statistic:  10.7 on 3 and 24 DF,  p-value: 0.0001181
> # Standardised beta coefficients
> standardCoefs(md_model2_MS)
                 b         beta
wcv    -0.01150839 -0.004632017
msfrac -1.94266204 -0.493399049
nffrac -1.35023562 -0.396747456
```

```
> ##########  MODEL 3: CONTROLS


> #
> #
> summary(ivf_model3_HC)
Call:
lm(formula = ivf ~ wcv + nffrac, data = data_controls)

Residuals:
      Min        1Q    Median        3Q       Max
-0.068467 -0.031470 -0.014156  0.001965  0.119724

Coefficients:
            Estimate Std. Error t value Pr(>|t|)
(Intercept) -0.06236    0.04354  -1.432    0.170
wcv          0.09689    0.06679   1.451    0.165
nffrac       0.19911    0.12189   1.634    0.121

Residual standard error: 0.05748 on 17 degrees of freedom
Multiple R-squared:  0.2078,    Adjusted R-squared:  0.1146
F-statistic:  2.23 on 2 and 17 DF,  p-value: 0.1381
> # Standardised beta coefficients
> standardCoefs(ivf_model3_HC)
                b      beta
wcv    0.09689124 0.3139253
nffrac 0.19910792 0.3535258



> #
> #
> summary(ndi_model3_HC)
Call:
lm(formula = ndi ~ wcv + nffrac, data = data_controls)

Residuals:
     Min       1Q   Median       3Q      Max
-0.21168 -0.09274  0.01848  0.09188  0.21352

Coefficients:
            Estimate Std. Error t value Pr(>|t|)
(Intercept)   1.0677     0.1094   9.758 2.22e-08 ***
wcv          -0.8058     0.1679  -4.801 0.000167 ***
nffrac       -0.1167     0.3063  -0.381 0.707835
---
Signif. codes:  0 ‘***’ 0.001 ‘**’ 0.01 ‘*’ 0.05 ‘.’ 0.1 ‘ ’ 1

Residual standard error: 0.1445 on 17 degrees of freedom
Multiple R-squared:  0.5755,    Adjusted R-squared:  0.5256
F-statistic: 11.52 on 2 and 17 DF,  p-value: 0.0006871
> # Standardised beta coefficients
> standardCoefs(ndi_model3_HC)
                b        beta
wcv    -0.8058089 -0.76049565
nffrac -0.1167391 -0.06037705
```

```
> #
> #
> summary(odi_model3_HC)
Call:
lm(formula = odi ~ wcv + nffrac, data = data_controls)

Residuals:
      Min        1Q    Median        3Q       Max 
-0.070917 -0.031150  0.006607  0.028346  0.123355 

Coefficients:
            Estimate Std. Error t value Pr(>|t|)    
(Intercept)  0.30462    0.03768   8.084 3.16e-07 ***
wcv          0.40821    0.05780   7.062 1.91e-06 ***
nffrac      -0.20131    0.10548  -1.909   0.0734 .  
---
Signif. codes:  0 ‘***’ 0.001 ‘**’ 0.01 ‘*’ 0.05 ‘.’ 0.1 ‘ ’ 1

Residual standard error: 0.04974 on 17 degrees of freedom
Multiple R-squared:  0.7662,    Adjusted R-squared:  0.7387 
F-statistic: 27.85 on 2 and 17 DF,  p-value: 4.321e-06
> # Standardised beta coefficients
> standardCoefs(odi_model3_HC)
                b       beta
wcv     0.4082054  0.8303175
nffrac -0.2013050 -0.2243938
```

```
> #
> #
> summary(fa_model3_HC)
Call:
lm(formula = fa ~ wcv + nffrac, data = data_controls)

Residuals:
     Min       1Q   Median       3Q      Max 
-0.23724 -0.05576 -0.01007  0.05907  0.12670 

Coefficients:
            Estimate Std. Error t value Pr(>|t|)    
(Intercept)  0.57018    0.07152   7.972 3.83e-07 ***
wcv         -0.49008    0.10972  -4.467 0.000339 ***
nffrac       0.06418    0.20021   0.321 0.752448    
---
Signif. codes:  0 ‘***’ 0.001 ‘**’ 0.01 ‘*’ 0.05 ‘.’ 0.1 ‘ ’ 1

Residual standard error: 0.09442 on 17 degrees of freedom
Multiple R-squared:  0.545,     Adjusted R-squared:  0.4914 
F-statistic: 10.18 on 2 and 17 DF,  p-value: 0.00124
> # Standardised beta coefficients
> standardCoefs(fa_model3_HC)
                 b        beta
wcv    -0.49007759 -0.73262958
nffrac  0.06417904  0.05257795
```

```
> #
> #
> summary(ad_model3_HC)
Call:
lm(formula = ad ~ wcv + nffrac, data = data_controls)

Residuals:
     Min       1Q   Median       3Q      Max 
-0.24276 -0.11443 -0.03911  0.06542  0.32769 

Coefficients:
            Estimate Std. Error t value Pr(>|t|)  
(Intercept)   0.1347     0.1367   0.986   0.3381  
wcv           0.5579     0.2097   2.660   0.0165 *
nffrac        0.5394     0.3827   1.410   0.1767  
---
Signif. codes:  0 ‘***’ 0.001 ‘**’ 0.01 ‘*’ 0.05 ‘.’ 0.1 ‘ ’ 1

Residual standard error: 0.1805 on 17 degrees of freedom
Multiple R-squared:  0.3353,	Adjusted R-squared:  0.2571 
F-statistic: 4.288 on 2 and 17 DF,  p-value: 0.03106
> # Standardised beta coefficients
> standardCoefs(ad_model3_HC)
               b      beta
wcv    0.5578856 0.5273671
nffrac 0.5393761 0.2794154
```

```
> #
> #
> summary(rd_model3_HC)
Call:
lm(formula = rd ~ wcv + nffrac, data = data_controls)

Residuals:
     Min       1Q   Median       3Q      Max 
-0.16102 -0.08164 -0.03465  0.03313  0.30887 

Coefficients:
            Estimate Std. Error t value Pr(>|t|)   
(Intercept) -0.05758    0.09914  -0.581  0.56898   
wcv          0.58944    0.15208   3.876  0.00121 **
nffrac       0.38720    0.27752   1.395  0.18091   
---
Signif. codes:  0 ‘***’ 0.001 ‘**’ 0.01 ‘*’ 0.05 ‘.’ 0.1 ‘ ’ 1

Residual standard error: 0.1309 on 17 degrees of freedom
Multiple R-squared:  0.4892,	Adjusted R-squared:  0.4292 
F-statistic: 8.142 on 2 and 17 DF,  p-value: 0.00331
> # Standardised beta coefficients
> standardCoefs(rd_model3_HC)
               b      beta
wcv    0.5894362 0.6734942
nffrac 0.3871988 0.2424498
```

```
> #
> #
> summary(md_model3_HC)
Call:
lm(formula = md ~ wcv + nffrac, data = data_controls)

Residuals:
     Min       1Q   Median       3Q      Max 
-0.17478 -0.06897 -0.04345  0.03244  0.28433 

Coefficients:
            Estimate Std. Error t value Pr(>|t|)   
(Intercept)  0.01832    0.10194   0.180  0.85951   
wcv          0.55625    0.15638   3.557  0.00242 **
nffrac       0.41344    0.28535   1.449  0.16556   
---
Signif. codes:  0 ‘***’ 0.001 ‘**’ 0.01 ‘*’ 0.05 ‘.’ 0.1 ‘ ’ 1

Residual standard error: 0.1346 on 17 degrees of freedom
Multiple R-squared:  0.4533,	Adjusted R-squared:  0.3889 
F-statistic: 7.047 on 2 and 17 DF,  p-value: 0.005904
> # Standardised beta coefficients
> standardCoefs(md_model3_HC)
               b      beta
wcv    0.5562520 0.6395313
nffrac 0.4134449 0.2604946



> ##########  MODEL 3: MS


> #
> #
> summary(ivf_model3_MS)
Call:
lm(formula = ivf ~ wcv + nffrac, data = data_multiple_sclerosis)

Residuals:
      Min        1Q    Median        3Q       Max 
-0.078300 -0.053158 -0.007364  0.020618  0.222168 

Coefficients:
            Estimate Std. Error t value Pr(>|t|)  
(Intercept)  0.09183    0.05314   1.728   0.0963 .
wcv         -0.01968    0.08583  -0.229   0.8205  
nffrac      -0.09811    0.11756  -0.835   0.4119  
---
Signif. codes:  0 ‘***’ 0.001 ‘**’ 0.01 ‘*’ 0.05 ‘.’ 0.1 ‘ ’ 1

Residual standard error: 0.07761 on 25 degrees of freedom
Multiple R-squared:  0.0273,	Adjusted R-squared:  -0.05052 
F-statistic: 0.3508 on 2 and 25 DF,  p-value: 0.7075
> # Standardised beta coefficients
> standardCoefs(ivf_model3_MS)
                 b        beta
wcv    -0.01968286 -0.04608362
nffrac -0.09811126 -0.16769736
```

```
> #
> #
> summary(ndi_model3_MS)
Call:
lm(formula = ndi ~ wcv + nffrac, data = data_multiple_sclerosis)

Residuals:
     Min       1Q   Median       3Q      Max 
-0.44633 -0.18572 -0.06742  0.26693  0.53608 

Coefficients:
            Estimate Std. Error t value Pr(>|t|)   
(Intercept)   0.3449     0.1911   1.805  0.08308 . 
wcv          -0.3321     0.3086  -1.076  0.29219   
nffrac        1.3294     0.4227   3.145  0.00425 **
---
Signif. codes:  0 ‘***’ 0.001 ‘**’ 0.01 ‘*’ 0.05 ‘.’ 0.1 ‘ ’ 1

Residual standard error: 0.2791 on 25 degrees of freedom
Multiple R-squared:  0.3387,	Adjusted R-squared:  0.2858 
F-statistic: 6.402 on 2 and 25 DF,  p-value: 0.005687
> # Standardised beta coefficients
> standardCoefs(ndi_model3_MS)
                b       beta
wcv    -0.3320733 -0.1782809
nffrac  1.3293665  0.5210322
```

```
> #
> #
> summary(odi_model3_MS)
Call:
lm(formula = odi ~ wcv + nffrac, data = data_multiple_sclerosis)

Residuals:
      Min        1Q    Median        3Q       Max 
-0.150518 -0.046022  0.001462  0.044308  0.104112 

Coefficients:
            Estimate Std. Error t value Pr(>|t|)    
(Intercept)  0.05502    0.04692   1.173 0.252011    
wcv          0.40893    0.07579   5.396 1.34e-05 ***
nffrac       0.39728    0.10381   3.827 0.000772 ***
---
Signif. codes:  0 ‘***’ 0.001 ‘**’ 0.01 ‘*’ 0.05 ‘.’ 0.1 ‘ ’ 1

Residual standard error: 0.06854 on 25 degrees of freedom
Multiple R-squared:  0.5983,	Adjusted R-squared:  0.5662 
F-statistic: 18.62 on 2 and 25 DF,  p-value: 1.119e-05
> # Standardised beta coefficients
> standardCoefs(odi_model3_MS)
               b      beta
wcv    0.4089258 0.6967726
nffrac 0.3972765 0.4941838
```

```
> #
> #
> summary(fa_model3_MS)
Call:
lm(formula = fa ~ wcv + nffrac, data = data_multiple_sclerosis)

Residuals:
     Min       1Q   Median       3Q      Max 
-0.31439 -0.19284  0.03288  0.10356  0.32339 

Coefficients:
            Estimate Std. Error t value Pr(>|t|)  
(Intercept)  0.18863    0.13555   1.392   0.1763  
wcv          0.01489    0.21894   0.068   0.9463  
nffrac       0.51868    0.29991   1.729   0.0961 .
---
Signif. codes:  0 ‘***’ 0.001 ‘**’ 0.01 ‘*’ 0.05 ‘.’ 0.1 ‘ ’ 1

Residual standard error: 0.198 on 25 degrees of freedom
Multiple R-squared:  0.1091,	Adjusted R-squared:  0.03786 
F-statistic: 1.531 on 2 and 25 DF,  p-value: 0.2359
> # Standardised beta coefficients
> standardCoefs(fa_model3_MS)
                b       beta
wcv    0.01488739 0.01307611
nffrac 0.51867560 0.33258661



> #
> #
> summary(ad_model3_MS)
Call:
lm(formula = ad ~ wcv + nffrac, data = data_multiple_sclerosis)

Residuals:
     Min       1Q   Median       3Q      Max 
-0.74795 -0.26388  0.07052  0.23271  0.73474 

Coefficients:
            Estimate Std. Error t value Pr(>|t|)    
(Intercept)   1.4108     0.2517   5.605 7.86e-06 ***
wcv          -0.1953     0.4065  -0.480 0.635110    
nffrac       -2.3258     0.5569  -4.177 0.000314 ***
---
Signif. codes:  0 ‘***’ 0.001 ‘**’ 0.01 ‘*’ 0.05 ‘.’ 0.1 ‘ ’ 1

Residual standard error: 0.3676 on 25 degrees of freedom
Multiple R-squared:  0.4124,	Adjusted R-squared:  0.3654 
F-statistic: 8.774 on 2 and 25 DF,  p-value: 0.001298
> # Standardised beta coefficients
> standardCoefs(ad_model3_MS)
                b        beta
wcv    -0.1953059 -0.07502971
nffrac -2.3257767 -0.65228129
```

```
> #
> #
> summary(rd_model3_MS)
Call:
lm(formula = rd ~ wcv + nffrac, data = data_multiple_sclerosis)

Residuals:
     Min       1Q   Median       3Q      Max 
-0.67285 -0.26975  0.00797  0.23831  0.91703 

Coefficients:
            Estimate Std. Error t value Pr(>|t|)    
(Intercept)   1.0560     0.2558   4.128 0.000357 ***
wcv          -0.1575     0.4132  -0.381 0.706307    
nffrac       -1.9936     0.5660  -3.522 0.001671 ** 
---
Signif. codes:  0 ‘***’ 0.001 ‘**’ 0.01 ‘*’ 0.05 ‘.’ 0.1 ‘ ’ 1

Residual standard error: 0.3737 on 25 degrees of freedom
Multiple R-squared:  0.3332,	Adjusted R-squared:  0.2799 
F-statistic: 6.246 on 2 and 25 DF,  p-value: 0.006309
> # Standardised beta coefficients
> standardCoefs(rd_model3_MS)
                b       beta
wcv    -0.1575034 -0.0634141
nffrac -1.9935676 -0.5859706
```

```
> #
> #
> summary(md_model3_MS)
Call:
lm(formula = md ~ wcv + nffrac, data = data_multiple_sclerosis)

Residuals:
     Min       1Q   Median       3Q      Max 
-0.69666 -0.22616  0.08983  0.18999  0.85450 

Coefficients:
            Estimate Std. Error t value Pr(>|t|)    
(Intercept)   1.1718     0.2470   4.745 7.22e-05 ***
wcv          -0.1617     0.3989  -0.405 0.688687    
nffrac       -2.1262     0.5464  -3.891 0.000655 ***
---
Signif. codes:  0 ‘***’ 0.001 ‘**’ 0.01 ‘*’ 0.05 ‘.’ 0.1 ‘ ’ 1

Residual standard error: 0.3607 on 25 degrees of freedom
Multiple R-squared:  0.379,	Adjusted R-squared:  0.3294 
F-statistic:  7.63 on 2 and 25 DF,  p-value: 0.002591
> # Standardised beta coefficients
> standardCoefs(md_model3_MS)
               b        beta
wcv    -0.161684 -0.06507626
nffrac -2.126182 -0.62474812
```

```
> ##########  MODEL 4: CONTROLS

> #
> #
> summary(ivf_model4_HC)
Call:
lm(formula = ivf ~ msfrac + nffrac, data = data_controls)

Residuals:
     Min       1Q   Median       3Q      Max 
-0.04887 -0.03827 -0.01734  0.00874  0.12060 

Coefficients:
            Estimate Std. Error t value Pr(>|t|)  
(Intercept) -0.11254    0.05536  -2.033    0.058 .
msfrac       0.39097    0.19567   1.998    0.062 .
nffrac       0.17024    0.11627   1.464    0.161  
---
Signif. codes:  0 ‘***’ 0.001 ‘**’ 0.01 ‘*’ 0.05 ‘.’ 0.1 ‘ ’ 1

Residual standard error: 0.05483 on 17 degrees of freedom
Multiple R-squared:  0.2791,	Adjusted R-squared:  0.1942 
F-statistic:  3.29 on 2 and 17 DF,  p-value: 0.06197
> # Standardised beta coefficients
> standardCoefs(ivf_model4_HC)
               b      beta
msfrac 0.3909676 0.4124886
nffrac 0.1702448 0.3022780



> #
> #
> summary(ndi_model4_HC)

Call:
lm(formula = ndi ~ msfrac + nffrac, data = data_controls)

Residuals:
    Min      1Q  Median      3Q     Max 
-0.3760 -0.1393  0.0372  0.1468  0.2626 

Coefficients:
            Estimate Std. Error t value Pr(>|t|)   
(Intercept)   0.7443     0.2238   3.325    0.004 **
msfrac       -0.0212     0.7911  -0.027    0.979   
nffrac       -0.0117     0.4701  -0.025    0.980   
---
Signif. codes:  0 ‘***’ 0.001 ‘**’ 0.01 ‘*’ 0.05 ‘.’ 0.1 ‘ ’ 1

Residual standard error: 0.2217 on 17 degrees of freedom
Multiple R-squared:  8.459e-05,	Adjusted R-squared:  -0.1176 
F-statistic: 0.0007191 on 2 and 17 DF,  p-value: 0.9993
> # Standardised beta coefficients
> standardCoefs(ndi_model4_HC)
                 b         beta
msfrac -0.02120068 -0.006515441
nffrac -0.01169630 -0.006049282
```

```
> #
> #
> summary(odi_model4_HC)
Call:
lm(formula = odi ~ msfrac + nffrac, data = data_controls)

Residuals:
     Min       1Q   Median       3Q      Max 
-0.13073 -0.06594 -0.03671  0.07443  0.18771 

Coefficients:
            Estimate Std. Error t value Pr(>|t|)    
(Intercept)  0.42701    0.09873   4.325  0.00046 ***
msfrac       0.19142    0.34897   0.549  0.59046    
nffrac      -0.26207    0.20736  -1.264  0.22335    
---
Signif. codes:  0 ‘***’ 0.001 ‘**’ 0.01 ‘*’ 0.05 ‘.’ 0.1 ‘ ’ 1

Residual standard error: 0.09779 on 17 degrees of freedom
Multiple R-squared:  0.0962,	Adjusted R-squared:  -0.01012 
F-statistic: 0.9048 on 2 and 17 DF,  p-value: 0.4233
> # Standardised beta coefficients
> standardCoefs(odi_model4_HC)
                b       beta
msfrac  0.1914243  0.1267920
nffrac -0.2620686 -0.2921266
```

```
> #
> #
> summary(fa_model4_HC)
Call:
lm(formula = fa ~ msfrac + nffrac, data = data_controls)

Residuals:
     Min       1Q   Median       3Q      Max 
-0.34531 -0.07387  0.02193  0.08139  0.18450 

Coefficients:
            Estimate Std. Error t value Pr(>|t|)   
(Intercept)   0.4957     0.1355   3.660  0.00194 **
msfrac       -0.5458     0.4788  -1.140  0.27005   
nffrac        0.1503     0.2845   0.528  0.60402   
---
Signif. codes:  0 ‘***’ 0.001 ‘**’ 0.01 ‘*’ 0.05 ‘.’ 0.1 ‘ ’ 1

Residual standard error: 0.1342 on 17 degrees of freedom
Multiple R-squared:  0.08117,	Adjusted R-squared:  -0.02693 
F-statistic: 0.7509 on 2 and 17 DF,  p-value: 0.487
> # Standardised beta coefficients
> standardCoefs(fa_model4_HC)
                b       beta
msfrac -0.5458467 -0.2657172
nffrac  0.1503371  0.1231619
```

```
> #
> #
> summary(ad_model4_HC)
Call:
lm(formula = ad ~ msfrac + nffrac, data = data_controls)

Residuals:
     Min       1Q   Median       3Q      Max 
-0.29422 -0.14149 -0.04616  0.15417  0.36413 

Coefficients:
            Estimate Std. Error t value Pr(>|t|)
(Intercept)   0.2129     0.2122   1.003    0.330
msfrac        0.6504     0.7500   0.867    0.398
nffrac        0.4401     0.4456   0.988    0.337

Residual standard error: 0.2102 on 17 degrees of freedom
Multiple R-squared:  0.09848,   Adjusted R-squared:  -0.007583 
F-statistic: 0.9285 on 2 and 17 DF,  p-value: 0.4143
> # Standardised beta coefficients
> standardCoefs(ad_model4_HC)
               b      beta
msfrac 0.6504072 0.2002085
nffrac 0.4400837 0.2279785
```

```
> #
> #
> summary(rd_model4_HC)
Call:
lm(formula = rd ~ msfrac + nffrac, data = data_controls)

Residuals:
     Min       1Q   Median       3Q      Max 
-0.17928 -0.11246 -0.03797  0.04113  0.43361 

Coefficients:
            Estimate Std. Error t value Pr(>|t|)
(Intercept)  0.01539    0.17431   0.088    0.931
msfrac       0.72891    0.61610   1.183    0.253
nffrac       0.28055    0.36609   0.766    0.454

Residual standard error: 0.1727 on 17 degrees of freedom
Multiple R-squared:  0.1111,    Adjusted R-squared:  0.00654 
F-statistic: 1.063 on 2 and 17 DF,  p-value: 0.3674
> # Standardised beta coefficients
> standardCoefs(rd_model4_HC)
               b      beta
msfrac 0.7289150 0.2712084
nffrac 0.2805472 0.1756684
```

```
> #
> #
> summary(md_model4_HC)
Call:
lm(formula = md ~ msfrac + nffrac, data = data_controls)

Residuals:
     Min       1Q   Median       3Q      Max 
-0.21117 -0.10256 -0.03048  0.09123  0.40454 

Coefficients:
            Estimate Std. Error t value Pr(>|t|)
(Intercept)  0.09503    0.17374   0.547    0.592
msfrac       0.65365    0.61408   1.064    0.302
nffrac       0.31423    0.36489   0.861    0.401

Residual standard error: 0.1721 on 17 degrees of freedom
Multiple R-squared:  0.1059,	Adjusted R-squared:  0.0007095 
F-statistic: 1.007 on 2 and 17 DF,  p-value: 0.3862
> # Standardised beta coefficients
> standardCoefs(md_model4_HC)
               b      beta
msfrac 0.6536466 0.2447161
nffrac 0.3142282 0.1979822




> ##########  MODEL 4: MS


> #
> #
> summary(ivf_model4_MS)
Call:
lm(formula = ivf ~ msfrac + nffrac, data = data_multiple_sclerosis)

Residuals:
     Min       1Q   Median       3Q      Max 
-0.08772 -0.04735 -0.02014  0.01904  0.23059 

Coefficients:
            Estimate Std. Error t value Pr(>|t|)   
(Intercept)  0.09012    0.03027   2.977  0.00638 **
msfrac      -0.17859    0.14438  -1.237  0.22760   
nffrac      -0.02524    0.12479  -0.202  0.84134   
---
Signif. codes:  0 ‘***’ 0.001 ‘**’ 0.01 ‘*’ 0.05 ‘.’ 0.1 ‘ ’ 1

Residual standard error: 0.07542 on 25 degrees of freedom
Multiple R-squared:  0.08147,	Adjusted R-squared:  0.007984 
F-statistic: 1.109 on 2 and 25 DF,  p-value: 0.3457
> # Standardised beta coefficients
> standardCoefs(ivf_model4_MS)
                 b        beta
msfrac -0.17858703 -0.26384795
nffrac -0.02524303 -0.04314682
```

```
> #
> #
> summary(ndi_model4_MS)
Call:
lm(formula = ndi ~ msfrac + nffrac, data = data_multiple_sclerosis)

Residuals:
     Min       1Q   Median       3Q      Max 
-0.35629 -0.12913 -0.02661  0.13628  0.40552 

Coefficients:
            Estimate Std. Error t value Pr(>|t|)    
(Intercept)  0.09275    0.08622   1.076 0.292288    
msfrac       1.79978    0.41117   4.377 0.000187 ***
nffrac       0.73366    0.35540   2.064 0.049508 *  
---
Signif. codes:  0 ‘***’ 0.001 ‘**’ 0.01 ‘*’ 0.05 ‘.’ 0.1 ‘ ’ 1

Residual standard error: 0.2148 on 25 degrees of freedom
Multiple R-squared:  0.6083,	Adjusted R-squared:  0.577 
F-statistic: 19.41 on 2 and 25 DF,  p-value: 8.166e-06
> # Standardised beta coefficients
> standardCoefs(ndi_model4_MS)
               b      beta
msfrac 1.7997810 0.6097277
nffrac 0.7336576 0.2875499
```

```
> #
> #
> summary(odi_model4_MS)
Call:
lm(formula = odi ~ msfrac + nffrac, data = data_multiple_sclerosis)

Residuals:
      Min        1Q    Median        3Q       Max 
-0.211085 -0.045019  0.003825  0.047606  0.265698 

Coefficients:
            Estimate Std. Error t value Pr(>|t|)    
(Intercept)   0.2500     0.0388   6.443 9.54e-07 ***
msfrac        0.2749     0.1850   1.486    0.150    
nffrac        0.1861     0.1599   1.164    0.255    
---
Signif. codes:  0 ‘***’ 0.001 ‘**’ 0.01 ‘*’ 0.05 ‘.’ 0.1 ‘ ’ 1

Residual standard error: 0.09666 on 25 degrees of freedom
Multiple R-squared:  0.201,	Adjusted R-squared:  0.1371 
F-statistic: 3.145 on 2 and 25 DF,  p-value: 0.06048
> # Standardised beta coefficients
> standardCoefs(odi_model4_MS)
               b      beta
msfrac 0.2749061 0.2955810
nffrac 0.1861389 0.2315437
```

```
> #
> #
> summary(fa_model4_MS)
Call:
lm(formula = fa ~ msfrac + nffrac, data = data_multiple_sclerosis)

Residuals:
      Min        1Q    Median        3Q       Max 
-0.271752 -0.166537  0.006372  0.142816  0.315352 

Coefficients:
            Estimate Std. Error t value Pr(>|t|)  
(Intercept)  0.16735    0.07509   2.229   0.0351 *
msfrac       0.62152    0.35809   1.736   0.0949 .
nffrac       0.27909    0.30952   0.902   0.3758  
---
Signif. codes:  0 ‘***’ 0.001 ‘**’ 0.01 ‘*’ 0.05 ‘.’ 0.1 ‘ ’ 1

Residual standard error: 0.1871 on 25 degrees of freedom
Multiple R-squared:  0.2048,    Adjusted R-squared:  0.1412 
F-statistic: 3.219 on 2 and 25 DF,  p-value: 0.05702
> # Standardised beta coefficients
> standardCoefs(fa_model4_MS)
               b      beta
msfrac 0.6215233 0.3444790
nffrac 0.2790916 0.1789599
```

```
> #
> #
> summary(ad_model4_MS)
Call:
lm(formula = ad ~ msfrac + nffrac, data = data_multiple_sclerosis)

Residuals:
     Min       1Q   Median       3Q      Max 
-0.46101 -0.15031 -0.02163  0.11149  0.59463 

Coefficients:
            Estimate Std. Error t value Pr(>|t|)    
(Intercept)   1.4223     0.1094  13.005 1.26e-12 ***
msfrac       -2.3866     0.5216  -4.576 0.000112 ***
nffrac       -1.3697     0.4508  -3.038 0.005506 ** 
---
Signif. codes:  0 ‘***’ 0.001 ‘**’ 0.01 ‘*’ 0.05 ‘.’ 0.1 ‘ ’ 1

Residual standard error: 0.2725 on 25 degrees of freedom
Multiple R-squared:  0.6773,    Adjusted R-squared:  0.6515 
F-statistic: 26.23 on 2 and 25 DF,  p-value: 7.248e-07
> # Standardised beta coefficients
> standardCoefs(ad_model4_MS)
               b       beta
msfrac -2.386566 -0.5785446
nffrac -1.369698 -0.3841419
```

```
> #
> #
> summary(rd_model4_MS)
Call:
lm(formula = rd ~ msfrac + nffrac, data = data_multiple_sclerosis)

Residuals:
     Min       1Q   Median       3Q      Max 
-0.47323 -0.26309  0.02437  0.15149  0.81643 

Coefficients:
            Estimate Std. Error t value Pr(>|t|)    
(Intercept)   1.0576     0.1311   8.068 2.01e-08 ***
msfrac       -1.7594     0.6252  -2.814  0.00939 ** 
nffrac       -1.2852     0.5404  -2.378  0.02535 *  
---
Signif. codes:  0 ‘***’ 0.001 ‘**’ 0.01 ‘*’ 0.05 ‘.’ 0.1 ‘ ’ 1

Residual standard error: 0.3266 on 25 degrees of freedom
Multiple R-squared:  0.4907,	Adjusted R-squared:  0.4499 
F-statistic: 12.04 on 2 and 25 DF,  p-value: 0.0002175
> # Standardised beta coefficients
> standardCoefs(rd_model4_MS)
               b       beta
msfrac -1.759403 -0.4469991
nffrac -1.285201 -0.3777600
```

```
> #
> #
> summary(md_model4_MS)
Call:
lm(formula = md ~ msfrac + nffrac, data = data_multiple_sclerosis)

Residuals:
     Min       1Q   Median       3Q      Max 
-0.46245 -0.18814 -0.03156  0.14488  0.74078 

Coefficients:
            Estimate Std. Error t value Pr(>|t|)    
(Intercept)   1.1799     0.1202   9.816 4.66e-10 ***
msfrac       -1.9453     0.5732  -3.394   0.0023 ** 
nffrac       -1.3462     0.4955  -2.717   0.0118 *  
---
Signif. codes:  0 ‘***’ 0.001 ‘**’ 0.01 ‘*’ 0.05 ‘.’ 0.1 ‘ ’ 1

Residual standard error: 0.2995 on 25 degrees of freedom
Multiple R-squared:  0.5721,	Adjusted R-squared:  0.5378 
F-statistic: 16.71 on 2 and 25 DF,  p-value: 2.467e-05
> # Standardised beta coefficients
> standardCoefs(md_model4_MS)
               b       beta
msfrac -1.945328 -0.4940762
nffrac -1.346216 -0.3955665
```

```
> ##########  MODEL 5: CONTROLS


> #
> #
> summary(ivf_model5_HC)
Call:
lm(formula = ivf ~ gfapfrac + ibafrac, data = data_controls)

Residuals:
      Min        1Q    Median        3Q       Max 
-0.067171 -0.033833 -0.010738  0.009946  0.132263 

Coefficients:
            Estimate Std. Error t value Pr(>|t|)  
(Intercept)   0.2236     0.1589   1.407   0.1775  
gfapfrac     -0.7025     0.4756  -1.477   0.1579  
ibafrac       1.3234     0.7179   1.844   0.0828 .
---
Signif. codes:  0 ‘***’ 0.001 ‘**’ 0.01 ‘*’ 0.05 ‘.’ 0.1 ‘ ’ 1

Residual standard error: 0.05691 on 17 degrees of freedom
Multiple R-squared:  0.2234,	Adjusted R-squared:  0.1321 
F-statistic: 2.446 on 2 and 17 DF,  p-value: 0.1166
> # Standardised beta coefficients
> standardCoefs(ivf_model5_HC)
                  b       beta
gfapfrac -0.7025421 -0.3191147
ibafrac   1.3233966  0.3982690



> #
> #
> summary(ndi_model5_HC)
Call:
lm(formula = ndi ~ gfapfrac + ibafrac, data = data_controls)

Residuals:
     Min       1Q   Median       3Q      Max 
-0.32435 -0.18018  0.08072  0.18435  0.24815 

Coefficients:
            Estimate Std. Error t value Pr(>|t|)
(Intercept)   0.5866     0.6051   0.969    0.346
gfapfrac      0.6468     1.8109   0.357    0.725
ibafrac      -2.3535     2.7333  -0.861    0.401

Residual standard error: 0.2167 on 17 degrees of freedom
Multiple R-squared:  0.04474,	Adjusted R-squared:  -0.06764 
F-statistic: 0.3981 on 2 and 17 DF,  p-value: 0.6777
> # Standardised beta coefficients
> standardCoefs(ndi_model5_HC)
                  b       beta
gfapfrac  0.6468128  0.0855807
ibafrac  -2.3534714 -0.2063090
```

```
> #
> #
> summary(odi_model5_HC)
Call:
lm(formula = odi ~ gfapfrac + ibafrac, data = data_controls)

Residuals:
     Min       1Q   Median       3Q      Max
-0.11748 -0.06645 -0.04244  0.06180  0.18434

Coefficients:
            Estimate Std. Error t value Pr(>|t|)
(Intercept)   0.2161     0.2806   0.770    0.452
gfapfrac      0.6284     0.8397   0.748    0.464
ibafrac      -0.7762     1.2674  -0.612    0.548

Residual standard error: 0.1005 on 17 degrees of freedom
Multiple R-squared:  0.04596,   Adjusted R-squared:  -0.06628
F-statistic: 0.4095 on 2 and 17 DF,  p-value: 0.6704
> # Standardised beta coefficients
> standardCoefs(odi_model5_HC)
                  b       beta
gfapfrac  0.6283600  0.1791870
ibafrac  -0.7761571 -0.1466424



> #
> #
> summary(fa_model5_HC)
Call:
lm(formula = fa ~ gfapfrac + ibafrac, data = data_controls)

Residuals:
     Min       1Q   Median       3Q      Max
-0.39948 -0.07792  0.05769  0.09133  0.13584

Coefficients:
             Estimate Std. Error t value Pr(>|t|)
(Intercept)  0.367684   0.384829   0.955    0.353
gfapfrac    -0.001882   1.151684  -0.002    0.999
ibafrac      1.259993   1.738286   0.725    0.478

Residual standard error: 0.1378 on 17 degrees of freedom
Multiple R-squared:  0.03059,   Adjusted R-squared:  -0.08346
F-statistic: 0.2682 on 2 and 17 DF,  p-value: 0.7679
> # Standardised beta coefficients
> standardCoefs(fa_model5_HC)
                    b          beta
gfapfrac -0.001882067 -0.0003944457
ibafrac   1.259992681  0.1749573932
```

```
> #
> #
> summary(ad_model5_HC)
Call:
lm(formula = ad ~ gfapfrac + ibafrac, data = data_controls)

Residuals:
     Min       1Q   Median       3Q      Max
-0.20988 -0.11719 -0.03551  0.12305  0.35821

Coefficients:
            Estimate Std. Error t value Pr(>|t|)
(Intercept)   0.7491     0.4789   1.564  0.13618
gfapfrac     -1.4098     1.4332  -0.984  0.33905
ibafrac       7.1961     2.1631   3.327  0.00399 **
---
Signif. codes:  0 ‘***’ 0.001 ‘**’ 0.01 ‘*’ 0.05 ‘.’ 0.1 ‘ ’ 1

Residual standard error: 0.1715 on 17 degrees of freedom
Multiple R-squared:  0.3997,	Adjusted R-squared:  0.3291
F-statistic: 5.661 on 2 and 17 DF,  p-value: 0.01306
> # Standardised beta coefficients
> standardCoefs(ad_model5_HC)
                 b       beta
gfapfrac -1.409845 -0.1868409
ibafrac   7.196123  0.6318458
```

```
> #
> #
> summary(rd_model5_HC)
Call:
lm(formula = rd ~ gfapfrac + ibafrac, data = data_controls)

Residuals:
     Min       1Q   Median       3Q      Max
-0.16217 -0.11006 -0.03879  0.04100  0.41924

Coefficients:
            Estimate Std. Error t value Pr(>|t|)
(Intercept)   0.5176     0.4685   1.105    0.285
gfapfrac     -1.0711     1.4020  -0.764    0.455
ibafrac       3.6584     2.1160   1.729    0.102

Residual standard error: 0.1678 on 17 degrees of freedom
Multiple R-squared:  0.1608,	Adjusted R-squared:  0.06206
F-statistic: 1.629 on 2 and 17 DF,  p-value: 0.2254
> # Standardised beta coefficients
> standardCoefs(rd_model5_HC)
                 b       beta
gfapfrac -1.071125 -0.1715812
ibafrac   3.658432  0.3882725
```

```
> #
> #
> summary(md_model5_HC)
Call:
lm(formula = md ~ gfapfrac + ibafrac, data = data_controls)

Residuals:
     Min       1Q   Median       3Q      Max 
-0.16473 -0.11381 -0.03856  0.04782  0.36762 

Coefficients:
            Estimate Std. Error t value Pr(>|t|)  
(Intercept)   0.5731     0.4381   1.308    0.208  
gfapfrac     -1.1156     1.3112  -0.851    0.407  
ibafrac       4.6881     1.9791   2.369    0.030 *
---
Signif. codes:  0 ‘***’ 0.001 ‘**’ 0.01 ‘*’ 0.05 ‘.’ 0.1 ‘ ’ 1

Residual standard error: 0.1569 on 17 degrees of freedom
Multiple R-squared:  0.2568,	Adjusted R-squared:  0.1693 
F-statistic: 2.936 on 2 and 17 DF,  p-value: 0.08028
> # Standardised beta coefficients
> standardCoefs(md_model5_HC)
                 b       beta
gfapfrac -1.115644 -0.1798243
ibafrac   4.688111  0.5006484



> ##########  MODEL 5: MS


> #
> #
> summary(ivf_model5_MS)
Call:
lm(formula = ivf ~ gfapfrac + ibafrac, data = data_multiple_sclerosis)

Residuals:
     Min       1Q   Median       3Q      Max 
-0.07925 -0.06235 -0.02322  0.02709  0.19149 

Coefficients:
            Estimate Std. Error t value Pr(>|t|)  
(Intercept)  0.12867    0.05783   2.225   0.0353 *
gfapfrac    -0.15683    0.14601  -1.074   0.2931  
ibafrac     -0.30979    0.32625  -0.950   0.3514  
---
Signif. codes:  0 ‘***’ 0.001 ‘**’ 0.01 ‘*’ 0.05 ‘.’ 0.1 ‘ ’ 1

Residual standard error: 0.07619 on 25 degrees of freedom
Multiple R-squared:  0.06258,	Adjusted R-squared:  -0.01242 
F-statistic: 0.8344 on 2 and 25 DF,  p-value: 0.4459
> # Standardised beta coefficients
> standardCoefs(ivf_model5_MS)
                  b       beta
gfapfrac -0.1568254 -0.2139386
ibafrac  -0.3097911 -0.1891433
```

```
> #
> #
> summary(ndi_model5_MS)
Call:
lm(formula = ndi ~ gfapfrac + ibafrac, data = data_multiple_sclerosis)

Residuals:
    Min      1Q  Median      3Q     Max 
-0.3533 -0.2251 -0.1042  0.1679  0.5839 

Coefficients:
            Estimate Std. Error t value Pr(>|t|)   
(Intercept)   0.2339     0.2264   1.033  0.31148   
gfapfrac      0.3137     0.5716   0.549  0.58802   
ibafrac       3.6311     1.2771   2.843  0.00877 **
---
Signif. codes:  0 ‘***’ 0.001 ‘**’ 0.01 ‘*’ 0.05 ‘.’ 0.1 ‘ ’ 1

Residual standard error: 0.2983 on 25 degrees of freedom
Multiple R-squared:  0.2447,	Adjusted R-squared:  0.1843 
F-statistic: 4.049 on 2 and 25 DF,  p-value: 0.02997
> # Standardised beta coefficients
> standardCoefs(ndi_model5_MS)
                b       beta
gfapfrac 0.313679 0.09812299
ibafrac  3.631139 0.50836712



> #
> #
> summary(odi_model5_MS)
Call:
lm(formula = odi ~ gfapfrac + ibafrac, data = data_multiple_sclerosis)

Residuals:
      Min        1Q    Median        3Q       Max 
-0.274107 -0.032878  0.008874  0.042744  0.299762 

Coefficients:
            Estimate Std. Error t value Pr(>|t|)    
(Intercept)  0.38953    0.07863   4.954  4.2e-05 ***
gfapfrac    -0.21406    0.19851  -1.078    0.291    
ibafrac      0.33593    0.44354   0.757    0.456    
---
Signif. codes:  0 ‘***’ 0.001 ‘**’ 0.01 ‘*’ 0.05 ‘.’ 0.1 ‘ ’ 1

Residual standard error: 0.1036 on 25 degrees of freedom
Multiple R-squared:  0.08232,	Adjusted R-squared:  0.008901 
F-statistic: 1.121 on 2 and 25 DF,  p-value: 0.3417
> # Standardised beta coefficients
> standardCoefs(odi_model5_MS)
                  b       beta
gfapfrac -0.2140628 -0.2125212
ibafrac   0.3359324  0.1492665
```

```
> #
> #
> summary(fa_model5_MS)
Call:
lm(formula = fa ~ gfapfrac + ibafrac, data = data_multiple_sclerosis)

Residuals:
     Min       1Q   Median       3Q      Max 
-0.24885 -0.20861  0.01768  0.14164  0.31518 

Coefficients:
            Estimate Std. Error t value Pr(>|t|)  
(Intercept)   0.2934     0.1452   2.021   0.0541 .
gfapfrac     -0.1507     0.3665  -0.411   0.6845  
ibafrac       1.6837     0.8190   2.056   0.0504 .
---
Signif. codes:  0 ‘***’ 0.001 ‘**’ 0.01 ‘*’ 0.05 ‘.’ 0.1 ‘ ’ 1

Residual standard error: 0.1913 on 25 degrees of freedom
Multiple R-squared:  0.1686,	Adjusted R-squared:  0.1021 
F-statistic: 2.535 on 2 and 25 DF,  p-value: 0.09943
> # Standardised beta coefficients
> standardCoefs(fa_model5_MS)
                  b        beta
gfapfrac -0.1506966 -0.07712194
ibafrac   1.6837183  0.38564986



> #
> #
> summary(ad_model5_MS)
Call:
lm(formula = ad ~ gfapfrac + ibafrac, data = data_multiple_sclerosis)

Residuals:
     Min       1Q   Median       3Q      Max 
-0.69936 -0.36150  0.09499  0.23024  0.89670 

Coefficients:
            Estimate Std. Error t value Pr(>|t|)    
(Intercept)   1.1488     0.2994   3.838 0.000751 ***
gfapfrac     -0.3062     0.7558  -0.405 0.688857    
ibafrac      -5.8018     1.6887  -3.436 0.002074 ** 
---
Signif. codes:  0 ‘***’ 0.001 ‘**’ 0.01 ‘*’ 0.05 ‘.’ 0.1 ‘ ’ 1

Residual standard error: 0.3944 on 25 degrees of freedom
Multiple R-squared:  0.3238,	Adjusted R-squared:  0.2698 
F-statistic: 5.987 on 2 and 25 DF,  p-value: 0.007509
> # Standardised beta coefficients
> standardCoefs(ad_model5_MS)
                  b        beta
gfapfrac -0.3061555 -0.06852901
ibafrac  -5.8017727 -0.58122246
```

```
> #
> #
> summary(rd_model5_MS)
Call:
lm(formula = rd ~ gfapfrac + ibafrac, data = data_multiple_sclerosis)

Residuals:
    Min      1Q  Median      3Q     Max 
-0.5218 -0.3001  0.0401  0.1815  1.0455 

Coefficients:
            Estimate Std. Error t value Pr(>|t|)   
(Intercept)   0.8912     0.2981   2.990  0.00619 **
gfapfrac     -0.4151     0.7527  -0.551  0.58619   
ibafrac      -5.0218     1.6817  -2.986  0.00624 **
---
Signif. codes:  0 ‘***’ 0.001 ‘**’ 0.01 ‘*’ 0.05 ‘.’ 0.1 ‘ ’ 1

Residual standard error: 0.3928 on 25 degrees of freedom
Multiple R-squared:  0.2634,	Adjusted R-squared:  0.2045 
F-statistic:  4.47 on 2 and 25 DF,  p-value: 0.02189
> # Standardised beta coefficients
> standardCoefs(rd_model5_MS)
                  b        beta
gfapfrac -0.4150926 -0.09737676
ibafrac  -5.0217878 -0.52725171
```

```
> #
> #
> summary(md_model5_MS)
Call:
lm(formula = md ~ gfapfrac + ibafrac, data = data_multiple_sclerosis)

Residuals:
     Min       1Q   Median       3Q      Max 
-0.57621 -0.32582  0.04304  0.19157  0.99413 

Coefficients:
            Estimate Std. Error t value Pr(>|t|)   
(Intercept)   0.9807     0.2916   3.363  0.00249 **
gfapfrac     -0.3955     0.7363  -0.537  0.59586   
ibafrac      -5.3158     1.6450  -3.231  0.00344 **
---
Signif. codes:  0 ‘***’ 0.001 ‘**’ 0.01 ‘*’ 0.05 ‘.’ 0.1 ‘ ’ 1

Residual standard error: 0.3842 on 25 degrees of freedom
Multiple R-squared:  0.2956,	Adjusted R-squared:  0.2393 
F-statistic: 5.247 on 2 and 25 DF,  p-value: 0.01251
> # Standardised beta coefficients
> standardCoefs(md_model5_MS)
                  b       beta
gfapfrac -0.3955338 -0.0927585
ibafrac  -5.3158165 -0.5579424
```
