# Supplementary material for: Neurite dispersion: a new marker of multiple sclerosis spinal cord pathology?
Source: Ann Clin Transl Neurol. 2017 Aug 15;4(9):663–79. doi: 10.1002/acn3.445 (PMC5590517; doi:10.1002/acn3.445)
Supplement: Supplementary file 4 — Data S4. Quantitative metrics in white matter peri‐plaque areas. [file ACN3-4-663-s004.pdf]

Supplementary material 4: quantitative metrics in white matter peri-plaque areas

Purpose

Focal white matter pathology in multiple sclerosis is usually identified following radiographical inspection of proton density-weighted and T2-weighted scans. However, the real burden of multiple sclerosis pathology goes beyond focal lesions, as pathology has been shown to extend from plaque borders. Here, we aim to characterise the alterations of the quantitative indices considered in this study in the surroundings of white matter plaques, measuring the dependence of histology-derived and MRI-derived metrics on the plaque distance.

Methods

Lesion delineation

Quantitative indices from histology (CV: circular variance, measuring neurite dispersion; MSF: myelin staining fraction; NSF: neurofilament staining fraction; ASF: astrocyte staining fraction; µGSF: microglia staining fraction), NODDI MRI (IVF: isotropic volume fraction, measuring free water content; NDI: neurite density index; ODI: orientation dispersion index) and DTI MRI (FA: fractional anisotropy; AD: axial diffusivity; RD: radial diffusivity; MD: mean diffusivity) were studied in areas surrounding white matter plaques, which were manually outlined on the mean b = 0 images. Histological indices were considered after co-registration to the MRI space, as explained in detail the main article.

Regression

Each voxel was characterised by its distance from the closest white matter lesion within the same MRI slice. Afterwards, the mean value of all metrics at a fixed discrete distance from the lesions was calculated. Lastly, the mean value of the metrics obtained as described above were regressed as a function of the distance from the lesion, i.e. fitting the linear model

$$m = a + b\,D,$$

where m is the generic histological/MRI metric and D is the distance from the lesion. The slope $b = \frac{dm}{dD}$ was considered significantly different from 0 if its associated p-value was smaller than 0.05. The analysis was performed in each multiple sclerosis specimen individually, and values of D up to 4000 µm were considered to fit the curve $m = m(D)$.

Results

Supplementary figures 4.1, 4.2 and 4.3 respectively show the experimental values of the histological, NODDI and DTI metrics as a function of the distance from the plaque. They also report the best linear fit, as well as the 95% fit prediction bounds. Statistically significant slopes are flagged by yellow text boxes. The figures show strong differences between the two multiple sclerosis specimens in the histological metrics, while MRI metrics show higher homogeneity between the two specimens. For example, in the upper thoracic case the astrocyte density ASF decreases as the distance from the lesion increases, while ASF increases in the upper

lumbar specimen. NODDI NDI shows a significant increase in both multiple sclerosis specimens as the distance from lesion varies, while IVF increases only in the upper thoracic specimen. Finally, DTI metrics AD, RD and MD all show decrease as a function of the distance from the lesions.

Discussion and conclusion

In this supplementary material we have shown histological and MRI (NODDI and DTI) metrics as a function of the distance from the lesions in the peri-plaque area. Results show variability of the trends in the histological metrics, likely due to the intrinsic variability of the histology methods. MRI metrics vary significantly as a function of the distance from the lesions, potentially highlighting the presence of a spatially variant ongoing disease process.

We conclude that quantitative MRI metrics as those considered in this study may be able to characterise pathology in the peri-plaque areas, although further work is required to precisely identify their histopathological correlates, due to the intrinsic variability of immunohistochemical labelling in characterising subtle changes over short spatial scales. In vivo, the limiting factor to characterise peri-plaque characteristics would be image resolution.

Histology

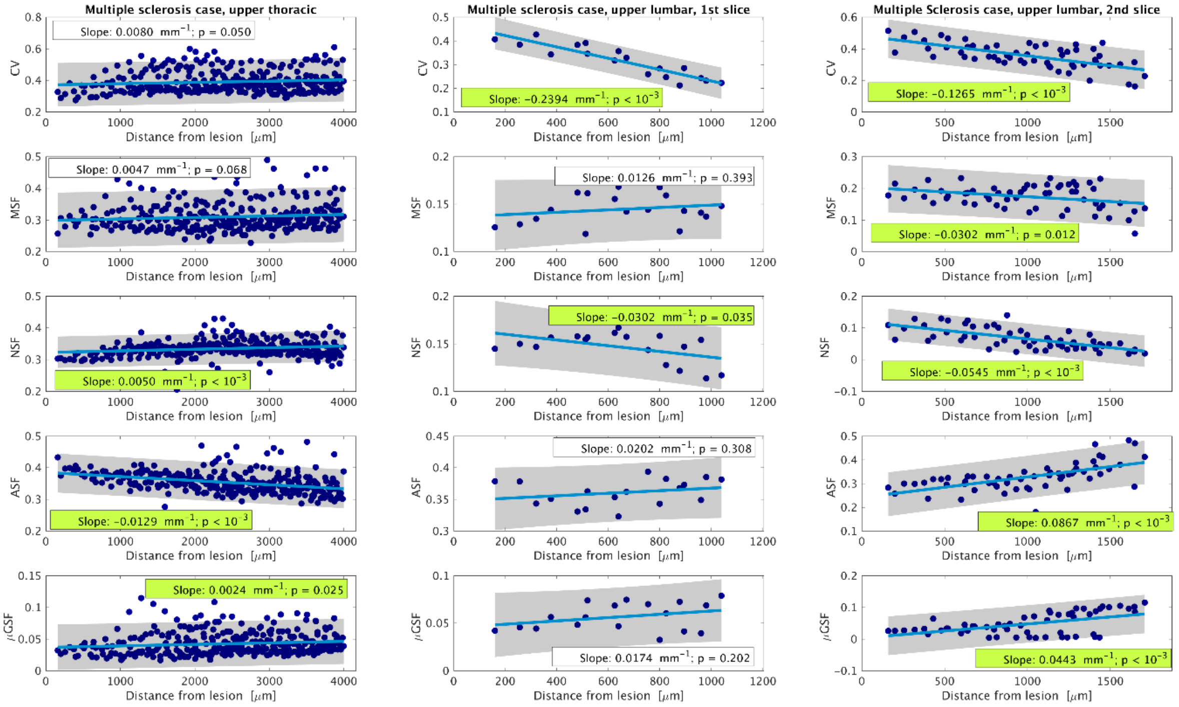

Supplementary figure 4.1: histological metrics as a function of the distance from the multiple sclerosis plaques. Left column: upper thoracic specimen; central column: upper lumbar specimen, first of the two MRI slices sampled by histology; left column: upper lumbar specimen, second of the two MRI slices sampled by histology.

NODDI

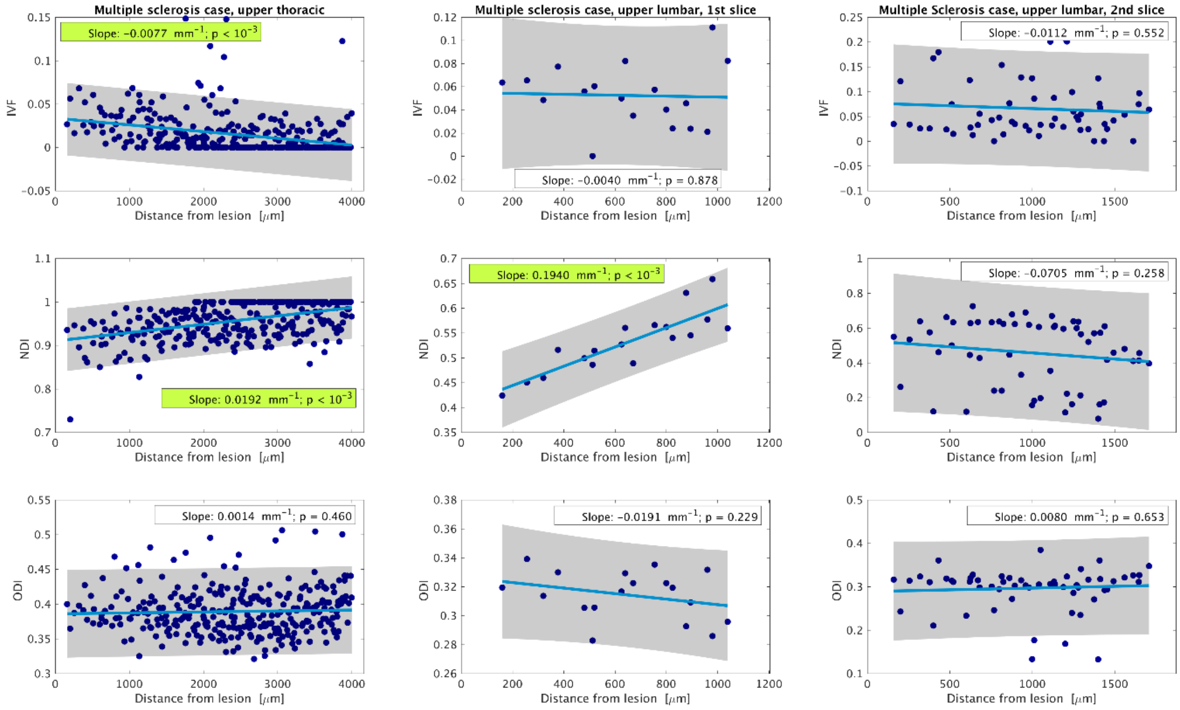

Supplementary figure 4.2: NODDI metrics as a function of the distance from the multiple sclerosis plaques. Left column: upper thoracic specimen; central column: upper lumbar specimen, first of the two MRI slices sampled by histology; left column: upper lumbar specimen, second of the two MRI slices sampled by histology.

DTI

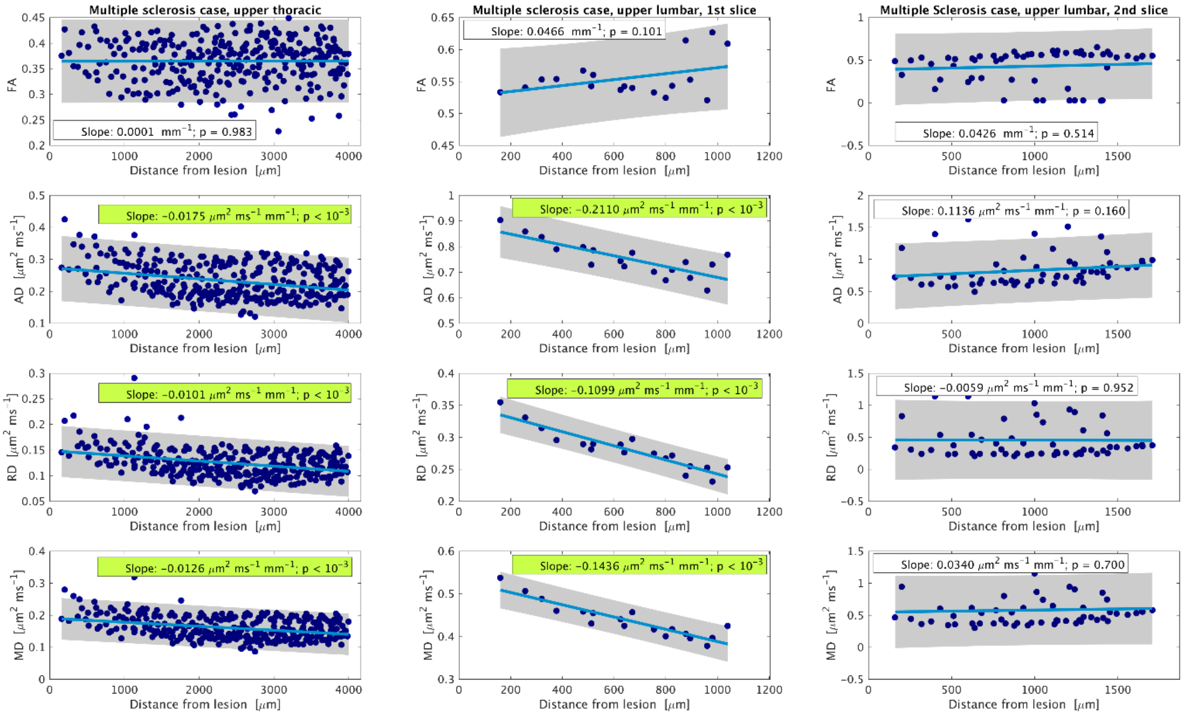

Supplementary figure 4.3: DTI metrics as a function of the distance from the multiple sclerosis plaques. Left column: upper thoracic specimen; central column: upper lumbar specimen, first of the two MRI slices sampled by histology; left column: upper lumbar specimen, second of the two MRI slices sampled by histology.
